# Endocrine Disrupting Chemicals Influence Hub Genes Associated with Aggressive Prostate Cancer

**DOI:** 10.3390/ijms24043191

**Published:** 2023-02-06

**Authors:** Diaaidden Alwadi, Quentin Felty, Changwon Yoo, Deodutta Roy, Alok Deoraj

**Affiliations:** 1 Department of Environmental Health Sciences, Florida International University, Miami, FL 33199, USA; 2 Department of Biostatistics, Florida International University, Miami, FL 33199, USA

**Keywords:** endocrine disruptive chemicals, prostate cancer, gene ontology, protein–protein interaction, molecular biomarkers, environmental health risk assessment

## Abstract

Prostate cancer (PCa) is one of the most frequently diagnosed cancers among men in the world. Its prevention has been limited because of an incomplete understanding of how environmental exposures to chemicals contribute to the molecular pathogenesis of aggressive PCa. Environmental exposures to endocrine-disrupting chemicals (EDCs) may mimic hormones involved in PCa development. This research aims to identify EDCs associated with PCa hub genes and/or transcription factors (TF) of these hub genes in addition to their protein–protein interaction (PPI) network. We are expanding upon the scope of our previous work, using six PCa microarray datasets, namely, GSE46602, GSE38241, GSE69223, GSE32571, GSE55945, and GSE26126, from the NCBI/GEO, to select differentially expressed genes based on |log2FC| (fold change) ≥ 1 and an adjusted *p*-value < 0.05. An integrated bioinformatics analysis was used for enrichment analysis (using DAVID.6.8, GO, KEGG, STRING, MCODE, CytoHubba, and GeneMANIA). Next, we validated the association of these PCa hub genes in RNA-seq PCa cases and controls from TCGA. The influence of environmental chemical exposures, including EDCs, was extrapolated using the chemical toxicogenomic database (CTD). A total of 369 overlapping DEGs were identified associated with biological processes, such as cancer pathways, cell division, response to estradiol, peptide hormone processing, and the p53 signaling pathway. Enrichment analysis revealed five up-regulated (NCAPG, MKI67, TPX2, CCNA2, CCNB1) and seven down-regulated (CDK1, CCNB2, AURKA, UBE2C, BUB1B, CENPF, RRM2) hub gene expressions. Expression levels of these hub genes were significant in PCa tissues with high Gleason scores ≥ 7. These identified hub genes influenced disease-free survival and overall survival of patients 60–80 years of age. The CTD studies showed 17 recognized EDCs that affect TFs (NFY, CETS1P54, OLF1, SRF, COMP1) that are known to bind to our PCa hub genes, namely, NCAPG, MKI67, CCNA2, CDK1, UBE2C, and CENPF. These validated differentially expressed hub genes can be potentially developed as molecular biomarkers with a systems perspective for risk assessment of a wide-ranging list of EDCs that may play overlapping and important role(s) in the prognosis of aggressive PCa.

## 1. Introduction

Prostate cancer (PCa) is the second leading cause of cancer death among men in the U.S. [1] and the world [2]. In the U.S., PCa is the most frequently diagnosed cancer (26% of all sites of cancer) and the second leading cause of death in men (11%) after lung cancer (22%) [3,4]. The standard diagnostic tools for PCa are prostate-specific antigen (PSA) serum levels, digital rectal examination, and biopsy [5]. PSA has been the most routinely utilized biomarker to screen and diagnose men for PCa [6]. However, PSA levels do not necessarily indicate PCa, which may be affected by different stimuli, such as inflammation or sexual activity, leading to overdiagnosis, overtreatment, and false-positive results [7,8]. Additionally, magnetic resonance imaging (MRI) has provided a significant advantage in the evaluation of PCa; however, it was discovered that MRI missed some intermediate and high-risk lesions of PCa [9]. The growth of PCa depends on androgens such as testosterone. These hormones interact with the androgen receptor (AR) whose dysregulated gene expression is linked to the development and aggressiveness of PCa. Since endocrine-disrupting chemicals (EDCs) found in the environment can mimic endogenous hormones, these environmental chemicals can activate molecular pathways involved in the growth and development of PCa. EDCs are associated with PCa’s poor prognosis [10,11,12,13,14,15]. Our earlier study showed the association of environmental phenols and parabens with patient-reported cases of PCa diagnoses [10]. In this study, we expand the scope of our work to include 22 environmental chemicals, especially EDCs, which are reported to influence PCa etiology in studies from the Chemical Toxicogenomic Database (CTD).

Microarray databases, high-throughput sequencing technology, and bioinformatics have played significant roles in the advancement of the medical field [10]. The Gene Expression Omnibus (GEO) online public microarray database allows analysis of differentially expressed genes (DEGs) that participate in biological processes (BP), cell components (CC), molecular functions (MF), gene regulatory networks, and pathways of PCa [7,15,16,17,18]. However, prior studies of DEGs’ analyses indicated proximate limitations, such as no reliable biomarker specified to differentiate tumors from normal tissues [19,20]. Additionally, most studies have concentrated on the differences in expression between various samples, and gene-to-gene interactions were mainly overlooked [15,16,17,18,19]. Single or multiple microarray datasets analysis in GEO has explored genes that play a significant role in the occurrence and progression of PCa, such as CASP5 and CASP8 [21], CDH1 and EPCAM [18], FOXO1 and NPM1 [19], TWIST1 and VEGFA [7], LMNB1 and ZWINT [22], IGF2 and KRT8 [23], PIK3R1 and ITGB4 [24], PPARG and PRKAR2B [25], CDCA8 and CDCA5 [26], and CYP3A4 and CYP3A7 [27]. However, the critical genes identified in these earlier studies are distinguishable from each other and have little in common, which can be attributed to the heterogeneous nature of the PCa [18].

Transcription factors (TF) bind to specific DNA sequences to regulate and control gene expressions. TFs are frequently altered in cancers due to DNA mutations, chromosomal abnormalities, chromatin amplifications, deletions, or landscape remodeling [28]. Therefore, a combination of several target gene expressions (TGE) can be used as a measure of TF signature activities [27]. TFs and gene regulators may impact the biological processes of cancer with their significant influence on biochemical pathways contributing to carcinogenesis. TFs such as AR, TWIST1, FOXA1, SOX9, E2F, and ASCL1 [28], or TATA, CEBPB, E2F, SRY, and NFKAPPAB [27], and modulators such as BUB1B, TOP2A, UBE2C, RRM2, and CENPF [28] have been indicated to be directly involved in biological processes creating numerous phenotypic modifications, proliferation, and differentiation in PCa progression. Furthermore, specific TFs targeting genes of PCa pathways may be acting as conduits to manifest the effects of the environmental chemicals and EDCs on the aggressive prognosis. Proteins are the functional entity of gene expressions and transcription activity. In a study, using the mining of proteomics studies, 41 differentially expressed proteins between cancer and normal or benign tissues were used to construct an extended PPI network related to PCa [29,30]. Nevertheless, such studies have not taken the influence of environmental chemicals into account to construct the PPI network. In our previous study, we used a risk assessment approach to demonstrate the association of environmental phenol and paraben exposures detected in urine samples, along with PCa, in U.S. men (NHANES data 2005–2015). We revealed a significant association of higher environmental phenols and parabens in the urine samples, categorical and numerical confounders, with self-reported PCa cases [10]. 

In this study, we used STRING database tools to construct the PPI network of the DEGs participating in PCa prognosis. We filtered the PPIs which appeared to be responsive to the chemicals, and the recognized EDCs that were evaluated for their potential contribution through the hub genes in the aggressiveness of PCa. 

Our research aimed to identify EDCs associated with PCa hub genes and/or transcription factors (TF) of these hub genes that influence molecular pathways in prostate carcinogenesis. The application of these PCa hub genes and/or their TFs as molecular biomarkers for risk assessments of EDCs may help in the prevention and control of PCa from exposure to EDCs found in the environment. 

## 2. Results

### 2.1. Screening of DEGs (Up- and Down-Regulated Genes)

**Figure 1:**
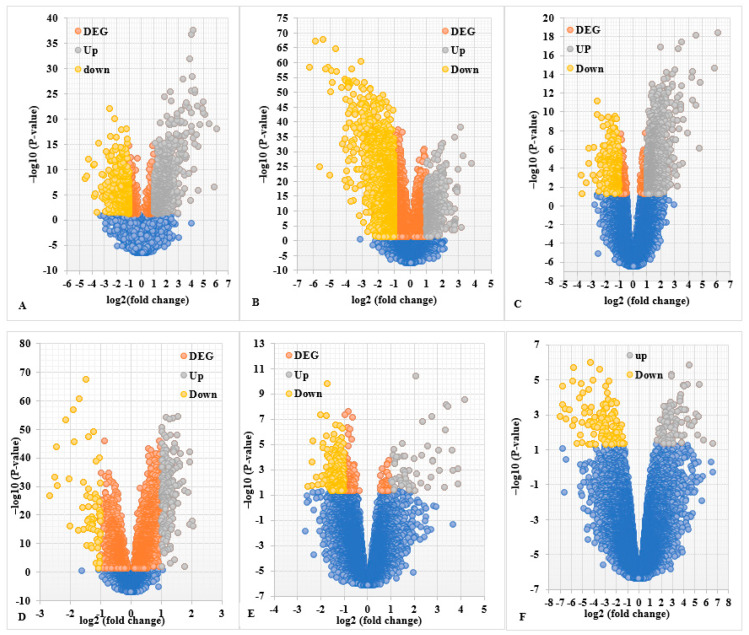
Identification of DEGs by volcano plot for PCa. Volcano plot for six GEO microarray datasets: (**A**): GSE46602, (**B**): GSE38241, (**C**): GSE69223, (**D**): GSE32571, (**E**): GSE55945, and (**F**): GSE26126. The criteria of DEGs cutoff standard are *p*-value < 0.05 and |log2FC| (fold change) > 1. Color code—gray: up-regulated genes; yellow: down-regulated genes; orange: no change in expression levels of DEGs; blue: non-DEGs).

**Figure 2:**
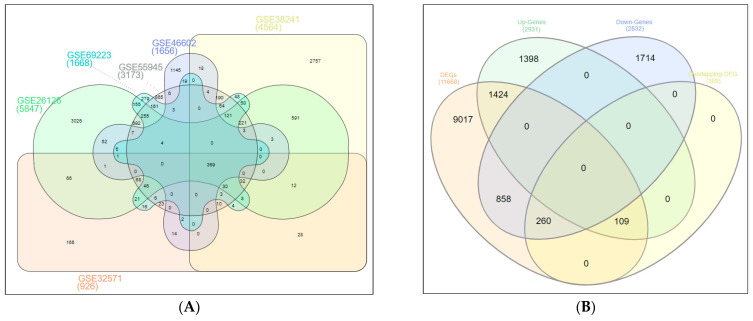
Venn diagram of the: (**A**) overlapping DEGs from six GEO microarray datasets for PCa, 369 DEGs were identified among the six GEO microarray datasets; and (**B**) DEGs (11668), up-regulated genes (2931), down-regulated genes (2832), and overlapping DEGs (369) from GEO microarray datasets for PCa. Overlapping up-regulated (109) and down-regulated (260) DEGs were identified. Color code.

Six PCa microarray datasets (GSE46602, GSE38241, GSE69223, GSE32571, GSE55945, and GSE26126) were analyzed using GEO2R online tools. We identified 2188, 6048, 2213, 1083, 3761, and 8300 DEGs, respectively, from each of the datasets. A total of 2832 up-regulated and 2931 down-regulated common genes from all six microarray datasets were downloaded. Standardization and normalization of the microarray datasets by GEO2R ensured that all selected samples had identical value distribution to determine the suitability of the study for further analysis and application of any queries. The gene expression profiles and data processing with the criteria of the cutoff standards of *p*-value < 0.05 and |log2FC| (fold change) > 1 are shown in Figure 1. The overlapping DEGs among six GEO microarray datasets included 369 genes, as illustrated in the Venn diagram (Figure 2A), and the overlapping down-regulated (260) and up-regulated (109) genes were identified (Figure 2B) for further analysis.

### 2.2. Gene Ontology Enrichment Analysis for DEGs in PCa

To associate the overlapping DEGs in PCa with biological functions, we performed gene ontology (GO) enrichment analyses, functional annotation, and pathway analysis using the Database for Annotation, Visualization, and Integrated Discovery (DAVID 6.8) online tool. This included the following three categories: BP (biological process), CC (cell component), and MF (molecular function). The top five pathway enrichment analyses for BP, CC, MF, and KEGG of up-regulated and down-regulated overlapping DEGs are shown in Tables S1 and S2. Down-regulated genes were markedly associated with BP (cell division, mitotic spindle organization, mitotic cytokinesis, mitotic cell cycle phase transition, and response to estradiol). For CC, down-regulated genes aligned with enriched cytosol, nucleoplasm, midbody, chromosome, centromeric region, and cyclin-dependent protein kinase holoenzyme complex. For MF, the down-regulated genes aligned with chromatin binding, protein binding, protein kinase binding, microtubule binding, and protein N-terminus binding. For the KEGG analysis, down-regulated DEGs were significantly enriched in the p53 signaling pathway, prostate cancer, apoptosis, and microRNAs involved in cancer pathways. The up-regulated DEGs were significantly involved in the BP of peptide hormone processing, long-chain fatty acid transport, regulation of gluconeogenesis, low-density lipoprotein particle remodeling, and calcium-mediated signaling. For CC, up-regulated genes aligned with extracellular exosome, neuronal cell body, basolateral plasma membrane, plasma membrane, and extracellular matrix. Additionally, the up-regulated genes in the MF aligned with oxidoreductase activity, long-chain fatty acid-CoA ligase activity, long-chain fatty acid transporter activity, structural molecule activity, and dicarboxylic acid transmembrane transporter activity. For the KEGG enrichment analysis, up-regulated DEGs were significantly associated with the calcium signaling pathway, metabolic pathways, Rap1 signaling pathway, and Ras signaling pathway. The TFs binding sites enrichment analysis of 109 up-regulated and 260 down-regulated genes showed overlapping DEGs in PCa by UCSC-TFBS. The analysis demonstrated the top ten TFs with the number of their target genes (Table 1). Nuclear transcription factor Y (NFY) with *p*-value = 6.69 × 10^−3^, FDR = 9.89 × 10^−1^, connected with (57 genes), was significantly associated with up-regulated genes. MAX Gene—MYC Associated Factor X (MYCMAX) with *p*-value = 5.35 × 10^−6^, FDR = 4.07 × 10^−4^, correlated with (133 genes), was significantly associated with down-regulated genes (Table 2). 

### 2.3. PPI Network Construction and Module Analysis

**Figure 3:**
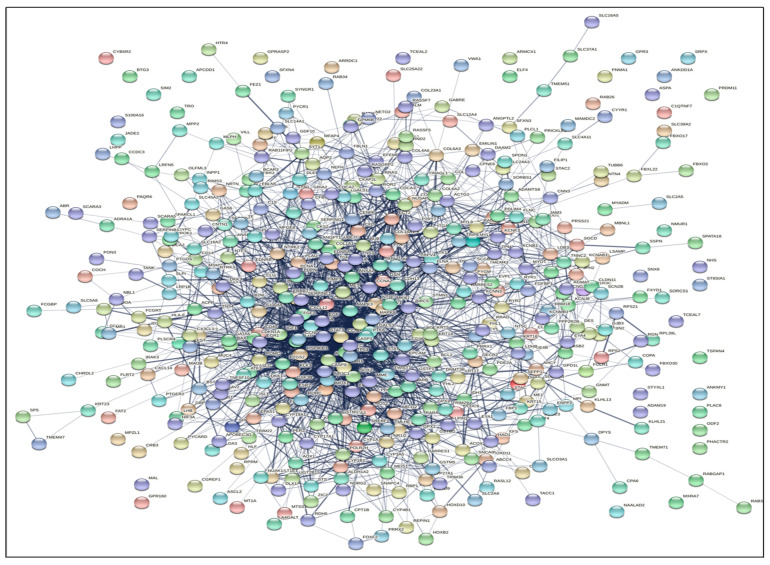
STRING database analysis of the PPI networks for the functional enrichment analysis yielded DEGs for 369 overlapping genes (nodes) and 2637 edges with degree > 11.2 and PP enrichment *p*-value < 1.0 × 10^−16^.

We conducted a functional analysis of the DEGs to construct the PPI network of the DEGs participating in PCa prognosis by utilizing the STRING database tool. There were 369 overlapping genes (nodes) and 2637 edges with a degree > 11.2, a clustering coefficient of 0.40, an average node degree of 5.53, and a PPI enrichment *p*-value < 1.0 × 10^−16^, which displayed the PPI enrichment for the network that was statistically significant as shown in Figure 3. Based on the PPI network, we used Cytoscape to identify hub genes among the 369 overlapping DEGs.

**Figure 4:**
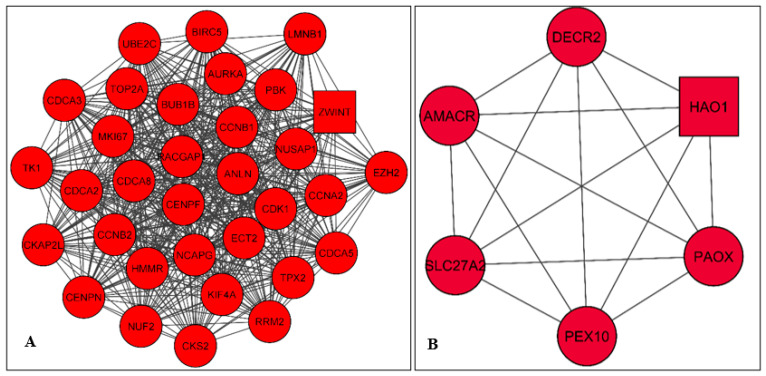
The two modules were generated from the DEGs PPI network by MCODE. (**A**) Module-1: associated with a score of 31.9, and includes 33 genes (nodes) and 511 edges. (**B**) Module-2: associated with a score of 6.0, consists of 6 genes (nodes) and 13 edges. Includes the top five clustered proteins (PEX10, SLC27A2, AMACR, PAOX, and DECR2), and one seed protein (HAO1) which is emphasized in the square node shape.

PPI network nodules were developed by employing MCODE (Figure 4). The DEGs generated two modules of the PPI network. Module-1 was associated with a score of 31.9 and included 33 genes (nodes) and 511 edges, and module-2 was associated with a score of 6.0, consisting of 6 genes (nodes) and 13 edges. The top six genes recognized in the module-2 network incorporated five clustered proteins (PEX10, SLC27A2, AMACR, PAOX, and DECR2), and one seed protein (HAO1), which was emphasized in the square-shaped node accountable for constructing the clusters. Additionally, we used the Cytohubba plugin of Cytoscape for classifying the top 20 genes (nodes) in the above PPI network according to four topological analysis techniques, including maximal clique centrality (MCC); degree, density of maximum neighborhood component (DNMC); and degree and edge percolated component (EPC) as shown in Table S4. Together, we identified 12 overlapping up-regulated (NCAPG, MKI67, CCNA2, CCNB1, TPX2) and down-regulated (CDK1, CCNB2, AURKA, UBE2C, BUB1B, CENPF, RRM) hub genes for subsequent analysis.

**Figure 5:**
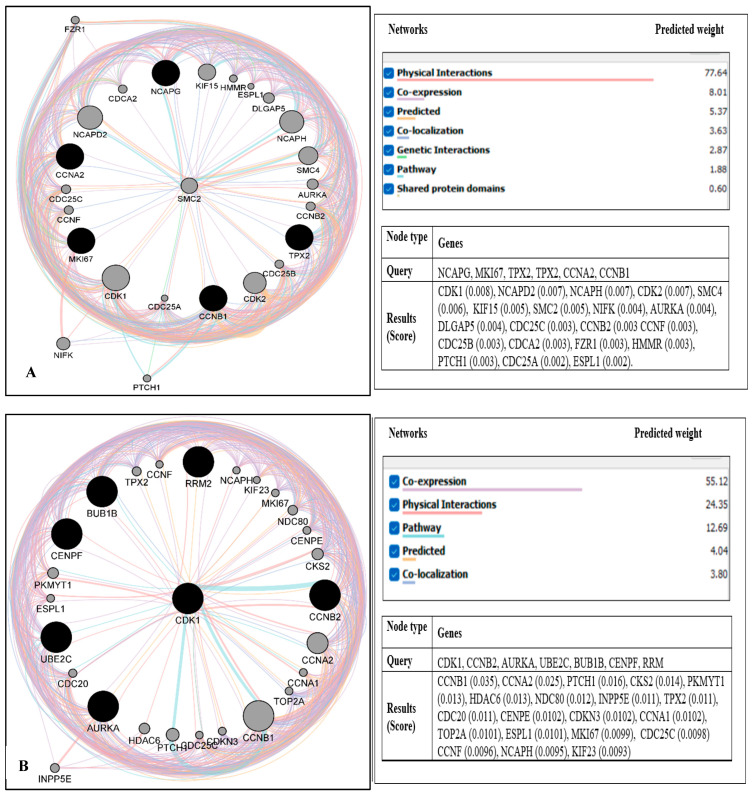
The network of hub genes (black circle), their related genes (gray circle), and the predicted weight percentages of the networks (physical interaction, co-expression, co-localization, genetic interaction, pathway, and shared protein domain) generated by GeneMANIA: (**A**) up-regulated hub genes with 25 genes were discovered with up-regulated genes and ordered with their score (circle size), and (**B**) down-regulated hub genes with 27 genes were discovered with down-regulated genes and ordered with their score (circle size).

A protein–gene interaction network for the hub genes and their effects on proteins/genes was developed through the GeneMANIA plugin of Cytoscape. The network of hub genes is shown in a black circle, and their related genes are shown in a gray circle (Figure 5). GeneMANIA revealed that 5 up-regulated hub genes were associated with 20 genes. The ranking order based on the score ranging from high to low is shown in Figure 5A. The predicted weight percentages of the up-regulated hub gene networks were as follows: physical interaction = 77.64, co-expression = 8.01, predicted = 5.37, co-localization = 3.63, genetic interaction = 2.87, pathway = 1.88, and shared protein domain = 0.6. GeneMANIA also demonstrated that 7 down-regulated hub genes were connected with 20 genes with the ranking order based on the score ranging from high to low as shown in Figure 5B. The predicted weight percentages of the down-regulated hub gene networks were as follows: co-expression = 55.12, physical interaction = 24.35, pathway = 12.69, predicted= 4.04, and co-localization = 3.8.

### 2.4. Dataset Validation for Expression of Hub Genes in PCa Tissues

**Figure 6:**
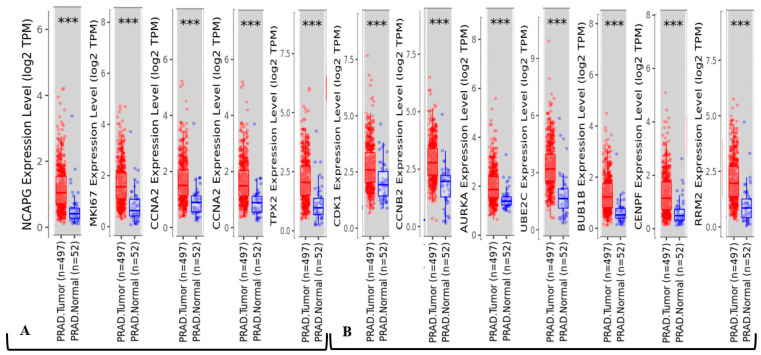
Differential analysis of expression of 12 hub genes between PCa (497) and normal prostate (52) tissues from Cancer Genome Atlas Prostate Adenocarcinoma (TCGA-PRAD) by TIMER: Tumor Immune Estimation Resource. (**A**): Query for 5 up-regulated hub genes. (**B**): Query for 7 down-regulated genes. ***: *p*-value < 0.001. TPM is transcripts per million with a normalization method for RNA-seq.

A full landscape of differential analysis of gene expression between PCa (497) and normal prostate (52) tissues from the RNA-Seq, TIMER database was performed based on the Cancer Genome Atlas Prostate Adenocarcinoma dataset (TCGA-PRAD) (Figure 6). Transcript per million (TPM) enrichment analysis was utilized to classify PCa-specific expression compared with normal tissues. It demonstrated the expression differences of 12 hub genes between PCa and normal prostate tissues. TPM value described the number of transcripts that were observed for a given gene or isoform. The results indicated that all hub genes (five up-regulated and seven down-regulated) were significantly changed in PCa tissues (*p*-value < 0.001) compared with the normal prostate.

Further, we used the UALCAN database to validate the transcript expression levels of 12 hub genes in PCa (492) and normal prostate tissues (52) from TCGA-PRAD. TPM enrichment analysis classified their expression in PCa. The threshold was set as |log2FC| (fold change) ≥ 1 and an adjusted *p*-value < 0.05. The results indicated for box whisker plots that all hub genes (up-regulated: NCAPG, MKI67, CCNA2, CCNB1, TPX2; and down-regulated: CDK1, CCNB2, AURKA, UBE2C, BUB1B, CENPF, RRM) were significantly changed in PCa tissues (*p*-value < 0.001) compared with the normal prostate tissues (Figure S1). The results demonstrated in box whisker plots that three up-regulated (NCAPG, MKI67, and CCNA2) and two down-regulated (CDK1, UBE2C) genes were significantly higher in PCa samples in patients aged 60–80 years compared with patients aged 41–60 years (*p*-value < 0.05) (Figure S2). It was also observed that hub genes (up-regulated: NCAPG, MKI67, CCNA2, CCNB1, TPX2; and down-regulated: CDK1, CCNB2, AURKA, UBE2C, BUB1B, CENPF, RRM) were significantly altered in PCa samples based on patients’ age (41–60 years and 60–80 years) (*p*-value < 0.05) compared with normal prostate tissues (Figure S2). Moreover, Figure S3 shows the positive relationship between hub genes and Gleason’s scores of the PCa samples. 

High expression levels of the 12 hub genes were associated with advanced stages (Gleason score ≥ 7) and recurrence, and the hub genes were significantly higher in the PCa tissues with the most aggressive stage (Gleason score = 10). Box whisker plot results showed that 10 hub genes (NCAPG, MKI67, CCNA2, CCNB1, TPX2, CDK1, AURKA, UBE2C, CENPF, RRM2) were significant (*p* < 0.001 or *p* < 0.01) with advanced stages (Gleason score ≥ 7) with the highest aggressiveness and the poorest prognosis. The other two hub genes (CCNB2, BUB1B) were also significant (*p* < 0.001 or *p* < 0.01 or *p* < 0.05) with advanced stages (Gleason score ≥ 7) and the most aggressive PCa with the poorest prognosis. 

Figure S4 shows the positive association between hub genes and the TP53 mutation status of the PCa samples. Box whisker plot results showed that 10 hub genes (NCAPG, MKI67, CCNA2, CCNB1, TPX2 CCNB2, AURKA, BUB1B, CENPF, RRM2) were significant (*p* < 0.001) with TP53-mutant status (n = 38) compared with the normal (n = 52) and nonmutant (295) samples. The other two hub genes (CDK1, UBE2C) were also significant (*p* < 0.001) with TP53-mutant status (n = 38) compared with the normal (n = 52) samples only. High expression levels of the 12 hub genes were associated with TP53-mutant status (n = 38) compared with the normal (n = 52) and nonmutant (295) samples.

**Figure 7:**
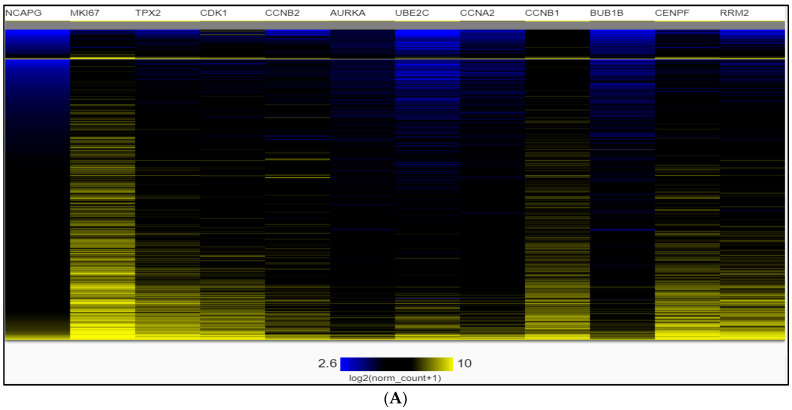

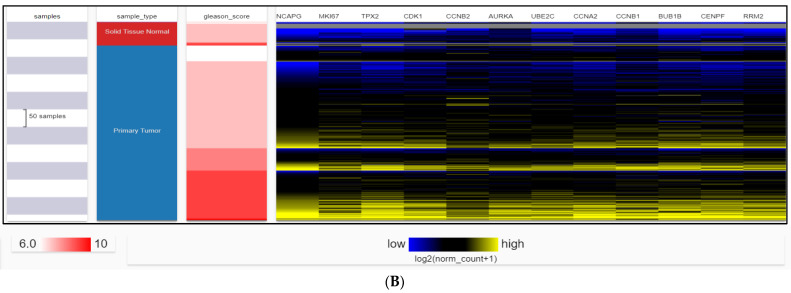
Heat map of 12 hub genes by UCSC-Xena. (**A**) The color parameters adjusted as max color 100% saturation of log2 (norm_count + 1) = 10.4 (yellow); min color 100% saturation of log2 (norm_count + 1) = 2.65 (blue) (**B**) The Gleason score was ranked from 5 (light pink) to 10 (dark pink). The cancer samples are shown in blue, and samples of pink are normal tissue. The high-expressed genes are marked yellow, and the low-expressed genes are blue (log2 (norm_count + 1).

The hierarchical clustering of the heatmap indicated that hub genes could distinguish PCa samples from noncancerous samples (Figure 7). The heatmap of the hub genes in PCa groups was significantly expressed compared with the normal groups by UCSC-Xena (Figure 7A,B). In addition, the hub genes had the nearest association to the Gleason score (Figure 7). We adjusted the most elevated color according to 100% saturation parameters of log2 (norm_count + 1) ≥ 10.4, and the lowest color according to 100% saturation parameters of log2 (norm_count + 1) ≤ 2.65 (Figure 7A). We compared solid normal tissue to primary tumor tissue (Figure 7B). The results showed that MKI67, TPX2, CDK1, CCNB2, UBE2C, CCNA2, CCNB1, BUB1B, CENPF, and RRM2 were overexpressed consistently in the TCGA-PRAD 568 samples by utilizing gene expression RNAseq–IlluminaHiSeq with a max of log2 (norm_count + 1) = 10.4 and min of log2 (norm_count + 1) = 2.65. Consequently, these hub genes were closely interconnected to PCa carcinogenesis and higher Gleason score stages.

### 2.5. Survival Analysis of Hub Genes

**Figure 8:**
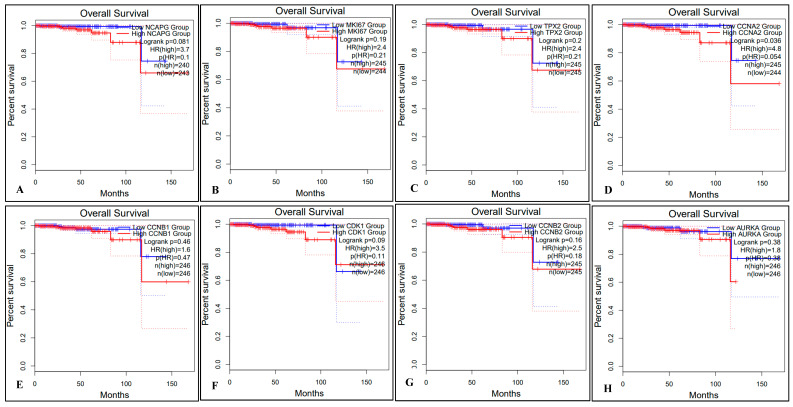

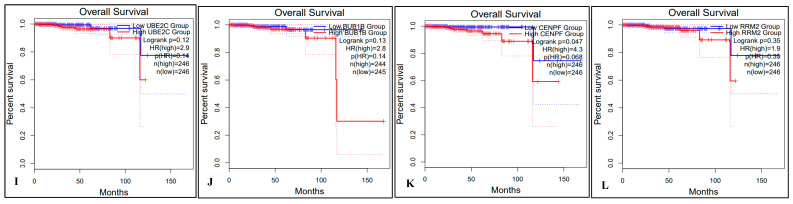
Prognostic survival analysis of the overall survival (OS) based on the high or low expression of 12 hub genes from TCGA in PCa patients, using the GEPIA online tool. OS analysis for 5 up-regulated (**A**–**E**) and 7 down-regulated (**F**–**L**) genes is presented. The dotted line on both sides of the curve represents the 95% confidence interval (95% CI), and log-rank *p* < 0.05 is considered as a statistically significant value. The red and blue lines represent high and low expressions of individual Hub genes (**A**–**L**), respectively in the PCa patient samples compared to the normal tissues.

**Figure 9:**
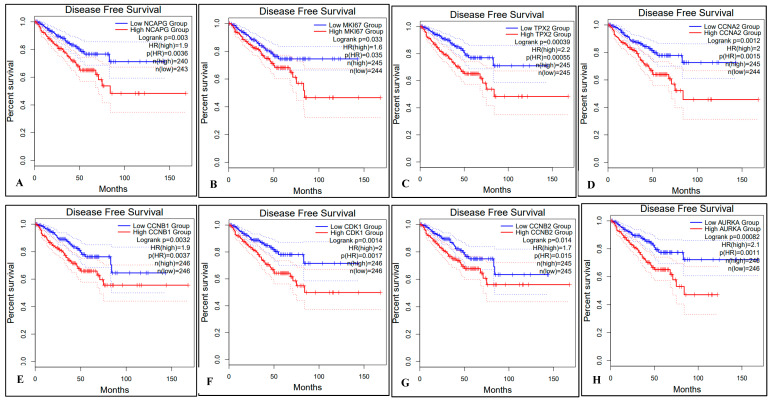

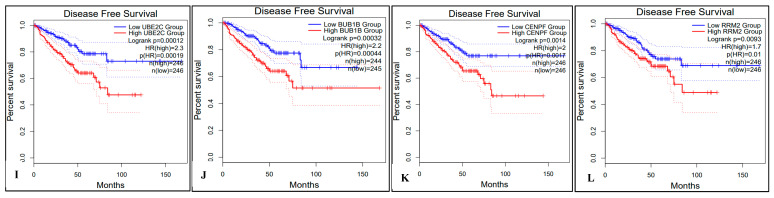
Prognostic survival analysis of DFS based on the high or low expression of 12 hub genes from TCGA in PCa patients was performed using GEPIA. OS analysis for 5 up-regulated (**A**–**E**) and 7 down-regulated (**F**–**L**) genes is presented. The dotted line on both sides of the curve represents the 95% confidence interval (95% CI), and log-rank *p* < 0.05 is considered as a statistically significant value. The red and blue lines represent high and low expressions of individual Hub genes (**A**–**L**), respectively in the PCa patient samples compared to the normal tissues.

We used the TCGA-PRAD modules in GEPIA2 to analyze the differential expression of the hub genes between PCa and normal tissues. To evaluate the association between hub genes and the progression of PCa, we performed overall survival (OS) and disease-free survival (DFS) analyses using the GEPIA online tools as shown in Figure 8. The results showed CCNA2 (up-regulated) and CENPF (down-regulated), was significantly influenced (*p*-value < 0.05) in the OS of the PCa patients (Figure 8D,K). The expression changes of all other genes (high or low) showed no effect on the OS of PCa patients (Figure 8A–C,E–J,L). We further applied survival analysis to evaluate DFS utilizing the GEPIA2 online database as shown in Figure 9. Cox regression analysis showed that DFS of PCa patients was significantly affected due to all up-regulated (NCAPG, MKI67, TPX2, CCNA2, CCNB1) and down-regulated (CDK1, CCNB2, AURKA, UBE2C, BUB1B, CENPF, RRM2) genes as shown in Figure 9A–E and Figure 9F–L, respectively.

**Figure 10:**
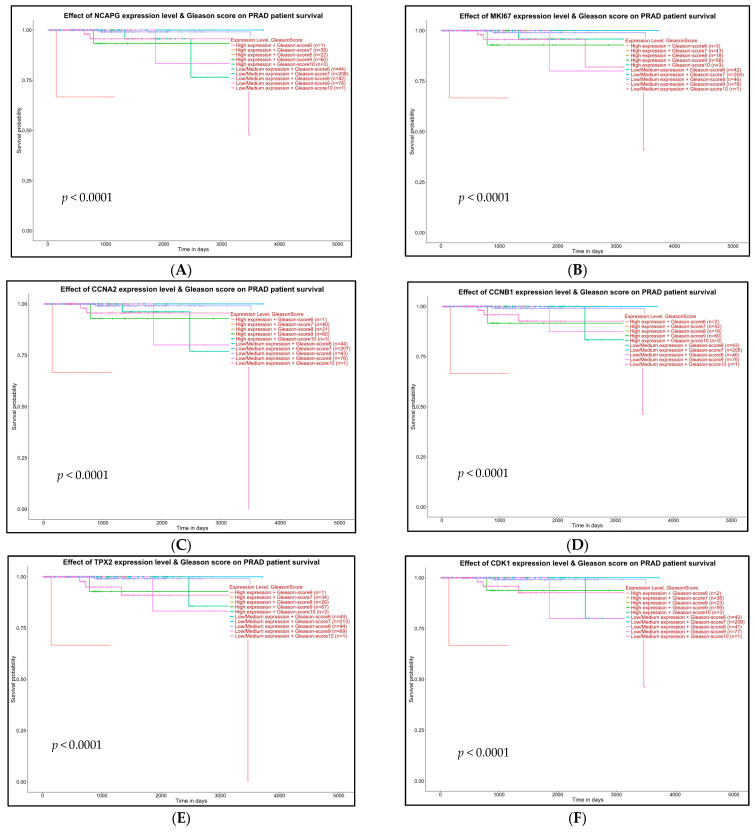

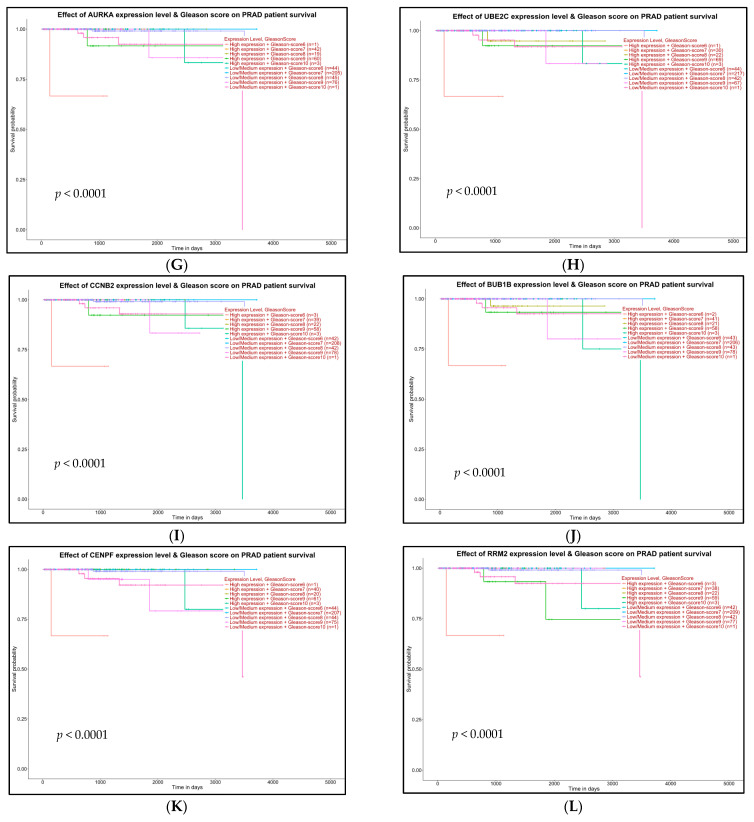
Predictive survival analysis based on the high or low expression of 12 hub genes from TCGA-PRDA in PCa patients was performed using the UALCAN. Survival analysis for 5 up-regulated (**A**–**E**) and 7 down-regulated (**F**–**L**) genes is presented. The survival analysis is based on the gene expression level and Gleason scores from the Cancer Genome Atlas Prostate Adenocarcinoma (TCGA-PRAD) patients’ survival. *p* < 0.05 is considered statistically significant.

The effect of gene expressions on the TCGA-PRAD patients’ survival based on their Gleason scores was performed using UALCAN. We demonstrated that all up-regulated (NCAPG, MKI67, TPX2, CCNA2, CCNB1) and all down-regulated (CDK1, CCNB2, AURKA, UBE2C, BUB1B, CENPF, RRM2) genes significantly affected (*p* < 0.001) the Gleason scores and TCGA-PRAD patients’ survival, as shown in Figure 10A–L.

### 2.6. Chemical-Gene Interaction Analysis for DEGs

**Figure 11:**
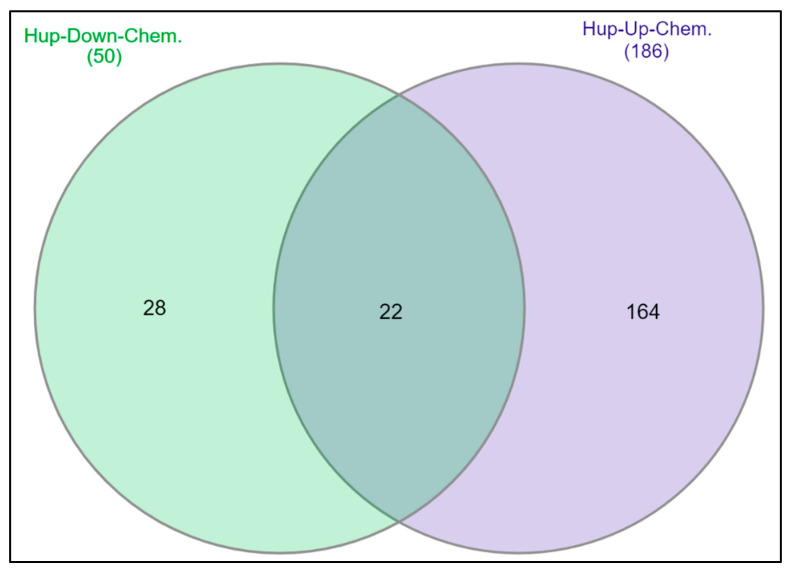
Venn diagram generated from CTD analyses of chemicals associated with 5 up-regulated hub genes (NCAPG, MKI67, TPX2, CCNA2, CCNB1) and 7 down-regulated hub genes (CDK1, CCNB2, AURKA, UBE2C, BUB1B, CENPF, RRM2) associated with PCa. Fifty chemicals are associated with 5 up-regulated and 186 with 7 down-regulated hub genes, and 22 chemicals have been shown to have effects on the expression levels of 12 hub genes.

We conducted analyses of the curated studies on the CTD for gene–disease connections, chemical–disease relationships, and chemical–gene interactions [31], and we utilized the PubMed database for cross-referencing. The Venn diagram (Figure 11) shows CTD analyses of chemicals that were associated with 5 up-regulated hub genes (NCAPG, MKI67, TPX2, CCNA2, CCNB1) and 7 down-regulated hub genes (CDK1, CCNB2, AURKA, UBE2C, BUB1B, CENPF, RRM2) associated with PCa in the CTD curated studies. There were 50 chemicals associated with the 5 up-regulated, and 186 chemicals associated with the 7 down-regulated hub genes. The overlapping 22 chemicals affecting the 12 hub genes were identified and listed in Table 3. Of the 22 identified chemicals, 17 were classified as recognized EDCs, and one chemical was carcinogenic. 

## 3. Discussion

PCa is a highly malignant cancer with complex molecular pathogenesis. The incomplete understanding of how environmental exposures to EDCs mimic hormones in the activation of cancer pathways is a critical barrier to the prevention and control of PCa. Long-term survival of PCa patients is still unsatisfactory due to delayed diagnosis, recurrence, medication resistance, and lack of understanding of the influence of environmental chemicals on the aggressiveness of PCa prognosis compounding these clinical gaps in the treatment of this disease. Despite the advances in the understanding of molecular pathology, PCa causes high morbidity and mortality in the male population [1,3,32]. In one of our earlier studies, we showed that environmental phenols and parabens were associated with patient-reported PCa with high Gleason scores through a set of hub genes [9]. Nonetheless, studies associating the risk of one or two chemical exposures for PCa or other cancers do not represent real-life scenarios where individuals are exposed to complex mixtures of many chemicals. In our study, we identified EDCs associated with PCa hub genes and/or TFs of these hub genes that play critical roles in the development and metastasis of PCa by the integration of gene microarray and RNA-seq datasets [15,24,25,27,33,34,35]. 

This study has expanded the scope of our earlier work in an attempt to include a comprehensive list of chemicals, especially EDCs, at least those for which information on their contribution to the molecular pathology of PCa prognosis or aggressiveness is available on curated databases (e.g., CTD or PubMed). In our first steps, we integrated and incorporated six PCa microarray studies from different groups and employed bioinformatics methods to examine gene expression profiles matching with clinical data from the TCGA and GEO databases. This study identified significant DEGs (11,668) and we selected up-regulated (2931) and down-regulated (2832) genes from the PCa studies. From this list, we picked overlapping (369) DEGs genes from all six GEO PCa microarray datasets. The number of down-regulated genes (260) was significantly higher than the number of up-regulated genes (109). To obtain a comprehensive view of the underlying molecular pathogenesis, it is important to focus on the gene networks that are participating in the PCa etiology as well as respond to environmental chemicals including EDCs. We used comprehensive bioinformatic tools including GO enrichment analysis of the selected 369 DEGs to understand how alterations in the expression of these genes may impact the biological pathways involved in the prognosis of PCa and patient survival. Our results indicated that the down-regulated DEGs were enriched in BP, including cell division, response to estradiol, and epithelial cell differentiation [36]. Cancer fibroblasts perform essential functions in cancer progression that involve inflammation and differentiation of cell division and epithelial cells [7]. For instance, the type 2 fibroblast growth factor receptor (FGFR2) blocked and intercepted prostate stem cell differentiation from the basal compartment cells and maintained stemness [37]. While the androgen receptor is the familiar target for PCa detection and therapy, more estrogens and their receptors have been involved in developing prostatic carcinoma [7,37]. In addition, cytokinesis or mitotic failure can potentially be the important mechanism contributing to the suffering of direct DNA damage [38]. During mitotic cytokinesis, weakening chromosomes are often partitioned into micronuclei, where they receive DNA damage in the following cell cycle, which builds cancer genomes by manipulating both numerical and structural alterations in chromosomes involved in tumor initiation and cancer progression [39,40]. Moreover, the KEGG pathway enrichment analysis of these down-regulated DEGs shows that they are involved in the main event of cancers, and prostate carcinoma including pathways in cancer, p53 signaling pathway, prostate cancer, apoptosis, and MicroRNAs in cancer. 

Our results then, indicated that the up-regulated DEGs were enriched in BP peptide hormone processing, long-chain fatty acid transport, oxidation–reduction process, low-density lipoprotein particle remodeling, and calcium-mediated signaling. Peptide hormones illustrate a primary category of hormones created from amino acids by specialized endocrine glands. However, an excessive amount of circulating peptide hormones is often connected with the presence of different tumors [41,42]. Metabolism deviation is a hallmark of cancer [42]. It is well documented that the cancerous cellular metabolisms are continuously encouraged to adapt to the increased proliferation rate and fulfill the nutritional needs to support the heightened cell division [42,43,44]. Similar to our results for PCa, one of the most noticeable metabolic changes is fatty acid or lipid metabolism [19,45,46]. Lipid biosynthesis is important for cell signaling and membrane formation. For example, the metabolic mediators of lipogenesis can operate as second messengers and influence PCa migration and invasion [47]. Additionally, the lipid metabolism of PCa is nearly connected to androgen by the androgen receptor (AR) signaling pathway. The AR signaling pathway can stimulate the uptake of exogenous lipids by PCa tissues and encourage adipose tissues to discharge fatty acids [48,49]. The oxidation–reduction process includes intracellular reactive oxygen species (ROS) and reactive nitrogen species (RNS). The most recognized enzymes generated by ROS and RNS are cyclooxygenases (COX) and lipoxygenases (LOX) [49]. Both COX and LOX mediate fatty acid metabolism, during which ROS are produced. The overexpression of COX and LOX in specific cancers indicates a possible involvement in carcinogenesis and progenesis in prostate carcinoma [50,51]. KEGG enrichment analysis reveals that up-regulated DEGs are affected in the main occurrence of cancer, including pathways in cancer, calcium signaling pathway [51], metabolic pathways [52], Rap1 signaling pathway [53], and Ras signaling pathway [54]. Therefore, all the above biological functions and pathways confirmed with our bioinformatics analysis results are closely interconnected to the development and progression of PCa.

We subjected the enrichment analyses to TFs binding sites of our identified 369 DEGs in the PCa pathway. The most significant TFs identified were nuclear transcription factor Y (NFY) for up-regulated, and MYC-associated factor X (MYCMAX) for down-regulated genes. Nuclear transcription factor Y (NFY) attaches to the CCAAT box, a component enriched in promoters of genes overexpressed in tumors [55]. NFY plays a major role in biochemical characterization of the target sequence of a DNA-binding matrix and various promoters, and NFY-regulated genes have a high density of biosynthetic pathways of purines and polyamines [56]. Examples of regulated genes AMD1 and ODC1 in several cancers, notably PCa, and ODC1, are needed for tumor appearance, and overexpression indicates patient survival [57]. While MYCMAX is an important regulator of growth in normal cells, it is also repeatedly connected with cancer progression, treatment resistance, and fatal outcomes in most human cancers [58]. Current conclusions have highlighted the potential significance of MYCMAX overexpression in the earliest phases of tumor-initiating cells and PCa formation. Various somatic genetic and epigenetic alterations in PCa cells, including loss of the tumor suppressors PTEN and p53, are connected to disease progression [59].

The research established on individual cancer types (such as PCa) recommended that genes often share the same functional pathway, therefore, the association between cancer modules and functional connectivity has been suggested to be investigated [60,61]. The two modules generated from the DEGs network in this study revealed module-1 with a score of 31.9 which included 33 nodes/genes and 511 edges, and module-2 with a score of 6.0, which included 6 nodes/genes, and 13 edges. In addition, the modules network of GeneMANIA predicted the 25 most significant up-regulated genes and 27 most significant down-regulated genes. 

Before we investigated the influence of EDCs on molecular pathology, it was important to determine the hub genes from our identified 369 DEGs which may have a pivotal role in the gene network of PCa prognosis. We applied STRING, MCODE, Cytohubba, and GeneMANIA analysis to determine five up-regulated (NCAPG, MKI67, TPX2, CCNA2, CCNB1) and seven down-regulated (CDK1, CCNB2, AURKA, UBE2C, BUB1B, CENPF, RRM2) hub genes. The full name and description of the 12 hub genes are listed in Table S3.

NCAPG has been indicated to be bonded to the overexpression of CCNB1. It is recommended to be a candidate target for hepatocellular carcinoma (HCC) therapy [62,63]. NCAPG is connected with NVAPG and functions as a target of miR-99a-3p in PCa cells. Overexpression is connected to castrate-resistant prostate cancers (CRPC), in which a sustained AR signal is considered the primary cause of CRPC [64]. It is associated with two markers for PCa: PSA for tumor cell differentiation and KI-67 for tumor cell proliferation and the epithelial–mesenchymal transition [65]. KI-67 may enhance the prognosis of PCa outcomes found on pathological parameters, enhancing the prognosis and monitoring of PCa subjects [64]. TPX2 is a microtubule-connected protein that targets TPX2-suppressed breast cancer by activating p53 and impeding the PI3k/AKT/P21 signaling pathways [66,67]. Lately, studies have demonstrated that targeting TPX2 in PCa reduced the rate of chromosome mis-segregation and, consequently, TPX2 is considered as a candidate biomarker for therapy [68]. TPX2 expression in PCa tissues was shown to be increased compared with normal tissues, and targeting TPX2 is a therapy strategy for PCa [66,67,68]. CCNA2 contributes to PCa invasion by modulating the expression of MMPs and VEGF and interacting with AR. CCNA2 is a cell cycle controller involved in the progression of PCa with metastatic activities, including VEGF and MMPs PCa [69]. Recent studies have demonstrated that CCNB1 particularly binds to CDC2 to increase cell migration, connected to the development of CRPC [70]. Higher levels of CCNB1 in PCa cells may have a beneficial effect on polyploidy and a prognostic biomarker for chemotherapy [70]. CDK1 controls mitochondrial metabolism for bioenergetics needed for tumor cell survival, and overexpression of CDK1 is associated with poor prognosis and metastasis in PCa [71]. CNB2 particularly binds CDC2 to improve cell migration which is connected to the development of CRPC and also plays a critical part in transforming growth factor beta-mediated cell cycle control [70,71]. UBE2C is key for the progression of PCa, and the level of UBE2C is important to predicting the prognosis of patients [72]. The AURKA gene has a crucial role in cell cycle development. Studies have indicated that it is correlated to the pathological stage and metastasis in HCC [73]. AURKA is a possible prognostic biomarker for the progression of high-risk small-cell PCa because it has been established to strengthen in 67% of PCa patients with highly aggressive hormone-naive castration-resistant cancer [74]. BUB1B is a critical mitotic checkpoint kinase identified as the top-scoring kinase by RNA interaction [75]. CENPF encodes a protein associated with the G2 phase, cell growth, protein synthesis, and the centromere–kinetochore complex and chromosomal segregation, and is related to aggressive prostate cancer [76]. RRM2 is an enzyme that specifies the rate of DNA synthesis and repair. Particularly, RRM2 was shown to be overexpressed in PCa patients with a high Gleason score and a progressive T stage and is considered a biomarker for PCa patients [77,78].

In our study, we conducted an extensive analysis to validate DEGs through TCGA-PRAD by using the UALCAN, TIMER, and UCSC-Xena databases. Our results indicated that all up-regulated (NCAPG, MKI67, CCNA2, CCNB1, TPX2) and down-regulated (CDK1, CCNB2, AURKA, UBE2C, BUB1B, CENPF, RRM2) genes were expressed significantly higher in PCa tissues compared with normal prostate tissues. In addition, up-regulated (NCAPG, MKI67, CCNA2) and down-regulated (CDK1, UBE2C) genes were highly expressed in older ages (61 years or older). A lower Gleason score (≤6) showed a better prognosis with no risk of metastasis, whereas an elevated Gleason score (>8) was associated with an increased risk of metastasis. All up-regulated (NCAPG, MKI67, CCNA2, CCNB1, TPX2) and down-regulated (CDK1, CCNB2, AURKA, UBE2C, BUB1B, CENPF, RRM2) genes were expressed significantly higher in aggressive PCa (Gleason score > 7) tissues. The hierarchical clustering of heatmap indicated that the hub genes could distinguish PCa samples from noncancerous ones. Heatmap also demonstrated that the hub genes in PCa groups were more significantly expressed than in normal groups. The results showed that MKI67, TPX2, CDK1, CCNB2, UBE2C, CCNA2, CCNB1, BUB1B, CENPF, and RRM2 were overexpressed consistently in the Cancer Genome Atlas Prostate Adenocarcinoma samples. Therefore, it appears that the DEGs validation through TCGA-PRAD for hub gene expressions in PCa samples from different datasets identifies NCAPG, MKI67, CCNA2, CDK1, and UBE2C as the unique hub genes associated with PCa carcinogenesis.

To further strengthen the relationship between hub genes and the progression of PCa, we performed OS and DFS using the GEPIA2 online database. The results showed that CCNA2 up-regulated and CENPF down-regulated genes significantly influenced the OS of the PCa patients. Utilizing TCGA-PRAD cohorts, all hub genes significantly influenced PCa patients’ DFS. Collectively, CCNA2 up-regulation and CENPF down-regulation were significantly associated with increased OS and DFS. To investigate the effect of hub gene expressions and Gleason scores we used the TCGA-PRAD patients’ survival and applied the UALCAN. The expression level and Gleason score were presented by high/low/medium expression + Gleason scores X (n). We demonstrated from patients’ survival analysis that there were positive relations with Gleason scores and hub genes. All hub genes (up-regulated: NCAPG, MKI67, TPX2, CCNA2, CCNB1; and down-regulated: CDK1, CCNB2, AURKA, UBE2C, BUB1B, CENPF, RRM2) affected the survival of the PCa patients with high Gleason scores (Figure 10). 

Interactions between EDCs and the PCa hub genes and/or proteins were determined using the CTD. These data are integrated with functional and pathway data to aid in the development of hypotheses about the mechanisms underlying environmentally influenced diseases [31]. We also utilized the PubMed database for cross-referencing. There were 50 chemicals associated with up-regulated, and 186 chemicals with down-regulated hub genes. In addition, of the overlapping 22 chemicals, 17 were classified as EDCs, and one chemical was carcinogenic (Table 3). Exposure to different EDCs may disrupt normal androgen and estrogen balance and possibly lead to sex hormone diseases [79,80,81,82]. Due to synergistic or additive effects, exposure to several chemicals in a mixture may be significant. These chemicals may have substantial impacts at lower concentrations than the NOAELS (no observed adverse effect levels) documented for individual chemicals [83,84]. The integrated toxicological effects of two or more mixtures can carry one of three conditions: dose addition, independent action, or interaction [85,86]. A meta-analysis study of persistent organic pollutants (POPs) was conducted regarding POP levels and the risk of PCa in the general population. POPs belong to EDCs and can be present in several food items. The study examined the PCa risk associated with each single and mixture of compounds [87]. Case-control studies showed positive linear and inverted U-shape associations between EDCs and the risk of PCa [88,89]. A case-control study revealed a positive association between plasma EDCs levels and metastatic PCa risk in Norwegians performed in 2015 [90]. Additionally, a positive association was discovered between high EDCs exposure among pesticide applicators and a positive history of PCa [91,92]. Of note is the fact that exposure to EDCs, primarily with estrogenic and androgenic actions, during embryonic evolution, at the various stages of susceptibility, can generate permanent changes that determine the tendency to PCa later in life. Using comprehensive bioinformatic tools and a CTD platform, the data presented here highlight the need for health risk assessment research on EDCs mixtures to sufficiently understand their function and influence on the molecular pathology of PCa. Taken together, our results here suggest that 17 EDCs from the chemical list (Table 4) affect the differential expressions of the discovered 12 hub genes. These genes, when aligned with their unique functional pathways, appear to influence the aggressiveness of PCa in patients with high Gleason scores.

EDCs can interrupt hormone synthesis and regular physiological functions of the male system. Most EDCs attach to nuclear hormone receptors (steroid hormone receptors), including ER and AR. As EDCs disrupt the activities of endogenous hormones, they may cause abnormal functions and stimulation of cancer growth (such as PCa) and dysfunctional immune and neuronal systems. EDCs can bind to ER and influence the transcription of target genes through genomic (transcriptional processes undergoing nuclear translocation) and non-genomic (passes signal transduction starting from steroid hormone receptors) pathways. Dysregulation of nuclear receptors is one mechanism by which EDCs may alter the expression of PCa hub genes contributing to the development or progression of PCa. In addition to nuclear receptor signaling, EDCs may also impact oxidative stress in cells. Oxidative stress can be a key regulator of EDC adverse effects on transcription regulation as many of these processes depend on redox reactions. Emerging studies describe TFs as target proteins of oxidative stress and hence EDCs-induced oxidative stress can be an essential regulator of TFs observed in the pathways identified previously. The biochemical pathways associated with EDCs may affect the ER-dependent signaling pathway. Exposure to EDCs has destructive effects on metabolism and endocrine and reproductive systems that can last for numerous generations [97,98].

There are limitations to our research. It was a predictive risk assessment of potential exposures of various EDCs and their effects on the aggressive PCa prognosis. All the data in the current study were based on the online mining of public databases for bioinformatics analysis and the data quality was not evaluated and assessed. Our results were restricted to selecting candidate hub genes connected with pathogenesis and PCa prognosis, which may have inadvertently overlooked some critical data. Nonetheless, the available datasets and tools presented the opportunity to conduct a thorough analysis to raise awareness and develop risk assessment methods to pave the way for further experimental validations of the identified 12 hub genes as biomarkers responsive to various EDCs exposures tested in this study for aggressive PCa prognosis.

## 4. Materials and Methods

**Figure 12:**
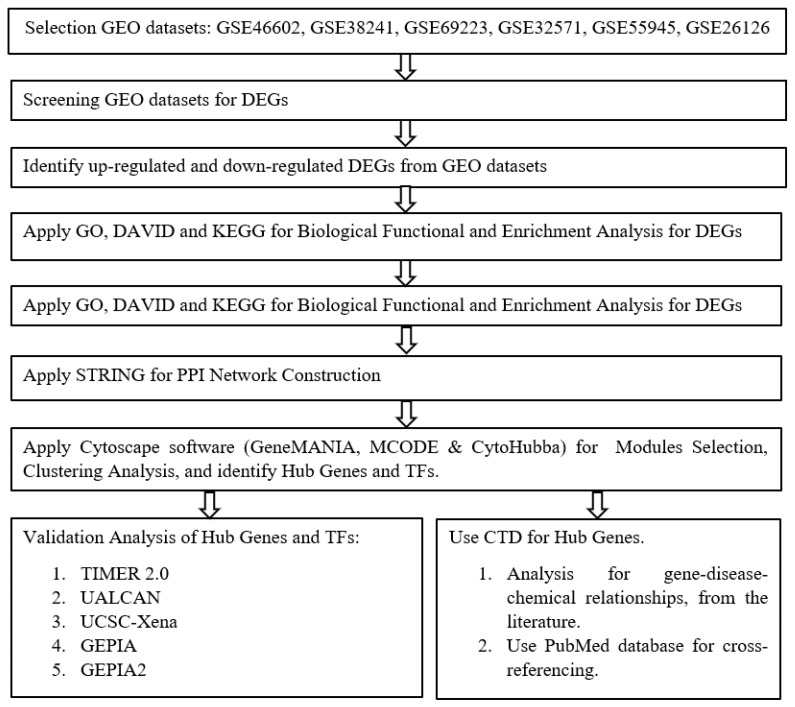
The flow chart depicts the steps to evaluate EDCs’ influence on the molecular prognosis and pathways of aggressive PCa by using different bioinformatic tools and datasets.

The steps to evaluate PCa molecular prognosis from GEO microarray datasets to identify the molecular and biological pathways, EDCs exposure (CTD), bioinformatics databases, gene set variation, and experimental validation analysis, are shown in a flow chart in Figure 12.

### 4.1. Microarray Datasets: Downloaded 

The datasets are associated with PCa from the NCBI/GEO database (https://www.ncbi.nlm.nih.gov/geo/, accessed on 15 January 2022) [99]. Six PCa gene expression microarray datasets GSE46602 [93], GSE38241 [94], GSE69223 [95], GSE32571 [96], GSE55945 [33], and GSE26126 [34], were identified, acquired, and downloaded from the NCBI/GEO. The characteristics of databases that were employed to detect the DEGs between PCa tissues and corresponding normal prostate tissues are shown in Table 4. Together, we analyzed 227 PCa tissues and 192 control samples. 

### 4.2. Data Processing: Screening and Identification of DEGs

The GEO2R (http://www.ncbi.nlm.nih.gov/geo/geo2r/, accessed on 15 January 2022) was used to screen DEGs between PCa samples and noncancerous control samples from six microarray datasets. GEO2R is a tool that permits researchers to identify DEGs by comparing different sample groups. The DEGs were screened and sorted, and the selection criteria were based on significance. The selection criteria for the DEGs were established on |log2FC| (fold change) ≥ 1 and an adjusted *p*-value < 0.05. The data of the individual microarrays and across the six datasets were used for the normalization of distribution. A Venn diagram was used to find the overlapping DEGs among the six microarray datasets. Volcano plots were used to indicate both median fold change and *p*-value using GraphPad Prism version 9.0 (GraphPad Software, Boston, MA, USA).

### 4.3. DEGs: GO, Biological Functional and Enrichment Analysis

DAVID.6.8 (https://david.ncifcrf.gov/, accessed on 30 February 2022) is a functional enrichment tool for high-throughput sequencing of gene datasets and proteomic research that provides biological, cellular, and molecular descriptions of a set of genes [100]. Kyoto Encyclopedia of Genes and Genomes (KEGG) was utilized for high-level and higher-order functions of cells and organisms of the biological system, molecular-level data generated by genome sequencing, and other high-throughput experimental technologies (https://www.genome.jp/kegg/, accessed on 10 March 2022) [101]. Biological process (BP), molecular function (MF), and cellular component (CC) analysis were examined in gene ontology (GO) (http://www.geneontology.org, accessed on 16 March 2022) [102] of the determined genes with the criterion for significance at a *p*-value < 0.05. Jointly, the GO and KEGG pathway analysis were employed to associate DEGs with their potential biological, molecular, and cellular functions and their processes in PCa pathways.

### 4.4. Protein–Protein Interaction (PPI) Network Construction 

Tool for the Retrieval of Interacting Genes (STRING) version 11.5 was implemented to construct an analysis of direct and indirect PPI networks. STRING is an online database tool (http://string-db.org/, accessed on 25 March 2022) that performs as an access point for interpreting relationships between diverse proteins on a genome-wide scale, which is beneficial for understanding protein interaction functions [103]. The analysis criteria conditions were human species, gene fusion databases, experiments, co-recurrence, local clustering coefficient 0.44, average node degree 7.59, PPI enrichment *p*-value < 1.0 × 10^−16^, and the minimum required interaction score of 0.4.

### 4.5. Modules Selection and Clustering Analysis 

Cytoscape software (version 3.9.0) was used for module analysis and selection. GeneMANIA, Molecular Complex Detection (MCODE), and CytoHubba were also used. MCODE was utilized to investigate the significant modules and select possible functional modules in the PPI network. MCODE parameters were: MCODE scores > 7, node score cutoff = 0.1, max depth = 100, degree cutoff = 2, and k-score = 2 [104]. CytoHubba is widely utilized to investigate the most significant node (genes) in different biological networks [105]. CytoHubba contains eleven topological analysis procedures for repeated measurements to reinforce the observation of the interactions. In our study, we conducted and included our results on MCC: maximal clique centrality; DNMC: degree, density of maximum neighborhood component; degree and EPC: degree and edge percolated component. GeneMANIA is a user-friendly web interface for investigating gene function, examining gene lists, and prioritizing genes for biological function. GeneMANIA extends the gene list with functionally similar genes that it specifies by utilizing functional genomics and proteomics data [106]. GeneMANIA was used to construct a molecular interaction network for DEGs, including co-expression networks, physical interaction, genetic interaction, co-localization pathway, and predicted and shared protein domain information [107].

### 4.6. External Dataset Validation and Evaluation of the Analysis of Hub Genes 

The hub genes identified and illustrated in the prognosis of PCa were validated on NCI’s Genomic Data Commons (TCGA-GDC), TCGA prostate cancer (TCGA-PRDA), and Prostate Adenocarcinoma (TCGS-PanCancer-Atlas). We applied a TIMER 2.0: Tumor Immune Estimation Response, to determine the differential gene expression analysis between PCa (492) and normal prostate (52) tissues. TIMER 2.0 (http://timer.cistrome.org/, accessed on 30 March 2022) is an online tool that provides a comprehensive resource for systematically analyzing immune infiltrates across different cancers. Validation of associations between gene expressions and tumor features in TCGA used immune estimations: expression profiles by TIMER, CIBERSORT, quanTIseq, xCell, MCP-counter, and EPIC algorithms [108] were studied. We then implemented the UALCAN (The University of Alabama at Birmingham Cancer data analysis Portal) database to investigate the expression of hub genes between PCa and normal samples based on: 1—sample types, 2—patients’ age, 3—patients’ Gleason score, and 4—TP53 mutation status, which is one of the most common genetic aberrations in cancer. UALCAN (http://ualcan.path.uab.edu/, accessed on 30 March 2022) is an interactive web resource that is user-friendly and widely used for analyzing cancer OMICS data. TP53 mutation status was acquired from TCGA whole-exome sequencing data by UALCAN, downloaded mutation annotation format (MAF) files (derived from VarScan2) from the Genomic Data Commons portal. The samples with/without TP53 mutation were matched with RNA-seq data. UALCAN accesses cancer OMICS data (TCGA, MET500, CPTAC, and CBTTC), and allows researchers to recognize biomarkers or conduct in silico validation of potential genes of interest [109]. At the University of California Santa Cruz (UCSC-Xena), online tools enable researchers to investigate functional genomic datasets for correlations between genomic and phenotypic variables [110]. Hierarchical clustering and heatmapping of hub genes were constructed by employing the UCSC-Xena (https://xenabrowser.net/, accessed on 30 March 2022). Highly expressed genes were calculated by (log2 (norm_count + 1), and Gleason score was ranked from 5 (light pink) to 10 (dark pink). 

### 4.7. Survival Analysis with Hub Genes 

UALCAN was used for prognostic survival analysis of the OS based on the high or low expression of hub genes from TCGA in PCa patients. The OS analysis is based on the effect of gene expression level and Gleason score on PCa (TCGA-PRDA) patients’ survival. To incorporate the heterogeneity of PCa samples at different stages of PCa progression among TCGA-PRAD patients, the gene expression levels and Gleason score were presented as follows:

High expression and Gleason score 6 (n)
High expression and Gleason score 7 (n)
High expression and Gleason score 8 (n)
High expression and Gleason score 9 (n)
High expression and Gleason score 10 (n)
Low/medium expression and Gleason score 6 (n)
Low/medium expression and Gleason score 7 (n)
Low/medium expression and Gleason score 8 (n)
Low/medium expression and Gleason score 9 (n)
Low/medium expression and Gleason score 10 (n)

The Gleason scores were categorized based on the risk as defined in Table S5. In addition, the online web GEPIA established on the TCGA database was applied for the OS and DFS of hub genes’ expression in PCa [111]. GEPIA2 is a revised version of GEPIA, created by a Peking University project team and qualified to examine the gene expression data of 9736 tumors and 8587 normal samples from TCGA and GTEx projects [29]. GEPIA and GEPIA2 perform OS and DFS analysis established on gene expression. GEPIA2 uses the log-rank test, at the Mantel–Cox test (Cox regression analysis), for the hypothesis test. The Cox regression analysis proportional hazard ratio and the 95% confidence interval information were included in the survival plots. The Kaplan–Meier (KM) OS and DFS (a 95% confidence interval (95% CI) and log-rank *p* < 0.05 was considered statistically significant) were employed for the evaluation of each hub gene’s prognostic value in PCa. We also used Gene Expression Profiling Interactive Analysis (GEPIA: http://gepia.cancer-pku.cn/index.html/, accessed on 30 March 2022 and GEPIA2: http://gepia2.cancer-pku.cn/#index/, accessed on 30 March 2022), an interactive web server, to analyze the comprehensive RNA sequencing expression data of genes from the TCGA and the GTEx projects, using a standard processing pipeline. 

### 4.8. Chemical-Gene Interaction Analysis for DEGs in PCa

To investigate the interaction between chemical exposure and differentially expressed hub genes in PCa, we performed the analysis using the manually curated research studies on the Comparative Toxicogenomic Database (CTD) [112]. We used this analysis to investigate the chemical–disease relationships, gene–disease connections, and chemical–gene interactions collected from the literature. We examined the PCa and discovered hub genes connected with EDCs. For the chemical–gene interaction query, we searched EDCs with PCa. Data showing curated association with the PCa, hub genes, and EDCs were downloaded, screened, sorted with studies that included only human samples, and cross-referenced using the PubMed database [31,113]. Transcription factors of the hub genes and their activity in response to EDC and other chemical exposure were identified by DAVID.6.8 (https://david.ncifcrf.gov/UCSC_TFBS/, accessed on 30 March 2022) [99]. 

### 4.9. Statistical Analysis

GEO2R was implemented to screen DEGs between PCa and normal tissue samples. GEO2R performs comparisons on original submitter-supplied processed data tables operating the GEO-query and linear models for microarray analysis (limma) R packages from the Bioconductor project. Bioconductor supplies access to statistical and graphical procedures for analyzing genomic data established on the R programming language. The GEO-query R package joins GEO data into R data structures that different R packages (Ver. 3.6.0) can use [113]. The limma R package was used as a statistical test for identifying DEGs, including normalization, background adjustment, and summarization [114]. The adjusted *p*-values and Benjamini and Hochberg FDR (false discovery rates) were used to balance the finding of statistically significant genes and to decrease the likelihood of false-positive errors.

## 5. Conclusions

The transcriptional expression levels of identified hub genes were significantly higher in PCa tissues of patients 60–80 years of age. Interestingly, all hub genes were associated with advanced stages (Gleason score ≥ 7) of PCa, suggesting their significant influence on the severity of PCa patients and their DFS. Further analysis using CTD revealed that 22 listed chemicals on CTD influence the selected hub genes in PCa prognosis. Seventeen of these chemicals are recognized EDCs and they specifically and significantly influence 6 of the 12 hub genes identified. We also delved into looking at the transcription factors (TF) of these identified 12 hub genes of the prostate cancer pathway considering that they may function as the conduit to the EDCs and other tested environmental chemicals’ effects in this study. What is striking about this research is that it uses comprehensive tools of bioinformatics to even include protein–protein interaction (PPI), which is the functional pathway of any gene transcription. The alignment of the identified 12 hub genes influenced by 22 chemicals (including 17 EDCs), with patient survival (PS), overall survival (OS), and disease-free survival (DFS), suggests that these hub genes potentially play a role(s) through various biological processes to contribute to the enhanced aggressiveness of prostate cancer in older patients. This observation suggests a significant influence of recognized EDCs on the molecular pathology of aggressive conditions in PCa patients and their disease-free survival. Combined and cumulative EDCs risk assessment on human health is very challenging [115], mostly due to the complexity of accurately extrapolating the effects, particularly when merging two or more EDCs with different toxicities. However, the validation of the 6 hub genes specific to EDC influences using UALCAN, UCSC-Xena, GEPIA2, and TCGA-PRDA strengthens the possibility of developing them as molecular biomarkers for EDC health risk assessments and early detection of prostate cancer aggressiveness in the older populations which may attain high Gleason scores > 7. 

## Figures and Tables

**Table 1:** UCSC_TFBS enrichment analyses showed TFs that putatively bind to the subsets of the genes by protein interactions option of the functional annotation tool of 109 up-regulated overlapping genes in PCa.

#	Term	Count	*p*-Value	FDR
1	NFY	57	6.69 × 10^−3^	9.89 × 10^−1^
2	CETS1P54	31	2.88 × 10^−3^	9.89 × 10^−1^
3	OLF1	50	3.99 × 10^−2^	9.89 × 10^−1^
4	SRF	73	6.28 × 10^−2^	9.89 × 10^−1^
5	COMP1	52	8.19 × 10^−2^	9.89 × 10^−1^
6	RP58	27	6.91 × 10^−1^	1.3 × 10^−2^
7	HMX1	21	1.23 × 10^−1^	1.99 × 10^−2^
8	NF1	30	1.32 × 10^−1^	1.99 × 10^−2^
9	PPARA	23	1.68 × 10^−1^	2.25 × 10^−2^
10	GFI1	22	6.7910^−1^	7.61 × 10^−2^

**Table 2:** UCSC_TFBS enrichment analysis showed TFs that bind to the subsets of the genes by the protein interactions option of the functional annotation tool of 260 down-regulated overlapping genes in PCa.

#	Term	Count	*p*-Value	FDR
1	MYCMAX	133	5.35 × 10^−6^	4.07 × 10^−4^
2	PAX4	179	7.63 × 10^−6^	4.07 × 10^−4^
3	PAX5	138	1.26 × 10^−5^	4.07 × 10^−4^
4	USF	142	1.27 × 10^−5^	4.07 × 10^−4^
5	NRSF	149	2.76 × 10^−5^	6.44 × 10^−4^
6	HEN1	140	3.02 × 10^−5^	6.44 × 10^−4^
7	P300	97	4.28 × 10^−5^	7.83 × 10^−4^
8	MAZR	61	1.55 × 10^−4^	2.37 × 10^−3^
9	AP4	148	1.83 × 10^−4^	2.37 × 10^−3^
10	NMYC	82	1.94 × 10^−4^	2.37 × 10^−3^

**Table 3:** The list of chemicals includes heavy metals, PAH, environmental phenols, pesticides, estrogenic compounds, and others. In this list, 17 are recognized EDCs that are associated with the hub genes in CTD studies.

	Chemical Name	Group	EDCs/Carcinogenic
1	Arsenic	Heavy metals	EDCs
2	Copper		EDCs
3	Cadmium		EDCs
4	Zinc		EDCs
5	Benzo(a)pyrene	Polycyclic aromatic hydrocarbons (PAH)	EDCs
6	Benzophenone-3	Environmental phenols	EDCs
7	Bisphenol A		EDCs
8	Methylparaben		EDCs
9	Propylparaben		EDCs
10	Sodium arsenate	Inorganic compounds	Carcinogenic
11	Copper sulfate		No
12	Dietary fats	Type of nutrient	No
13	Diethylstilbestrol	Synthetic (manufactured) form of estrogen	EDCs
14	Dihydrotestosterone	Steroid hormone	No
15	Testosterone		No
16	Estradiol	Estrogenic steroid	EDCs
17	Genistein	Polyphenolic isoflavone	EDCs
18	DDT	Pesticides	EDCs
19	Heptachlor		EDCs
20	Aldrin		EDCs
21	Chlordecone		EDCs
22	Phthalates	Polyvinyl chloride (PVC)/plasticizers	EDCs

**Table 4:** Characteristics of the selected GEO microarray dataset profiles associated with PCa.

GEO Profile	Case	Control	Platform	Annotation Platform	References
GSE46602	36	14	GPL570	Affymetrix Human Genome U133 Plus 2.0 Array	[93]
GSE38241	18	21	GPL4133	Agilent-014850 Whole Human Genome Microarray	[94]
GSE69223	15	15	GPL570	Affymetrix Human Genome U133 Plus 2.0 Array	[95]
GSE32571	95	39	GPL6947	Illumina HumanHT-12 V3.0 expression BeadChip	[96]
GSE55945	4	4	GPL570	Affymetrix Human Genome U133 Plus 2.0 Array	[33]
GSE26126	95	98	GPL8490	Illumina HumanMethylation27 BeadChip	[34]
Total	227	191			

## Data Availability

The datasets generated during the current study are available from the corresponding author upon reasonable request.

## Supplementary Materials

The following supporting information can be downloaded at: https://www.mdpi.com/article/10.3390/ijms24043191/s1.

## Abbreviations

AR	Androgen receptor
AURKA	Aurora kinase A
BP	Biological process
BUB1B	BUB1 mitotic checkpoint serine/threonine kinase B
CRPC	Castrate-resistant prostate cancers
CC	Cellular component
CCNA2	Cyclin A2
CCNB1	Cyclin B1
CCNB2	Cyclin B2
CDK1	Cyclin dependent kinase 1
COX	Cyclooxygenases
CENPF	Centromere protein F
CTD	Comparative Toxicology Database
DEGs	Differentially expressed genes
DNMC	Degree, density of maximum neighborhood component
EDCs	Endocrine-disrupting chemicals
EPC	Edge percolated component
FDR	False discovery rates
GEO	Gene expression omnibus
GO	Gene ontology
KEGG	Kyoto Encyclopedia of Genes and Genomes
LOX	Lipoxygenases
MCODE	Molecular complex detection
MYCMAX	MAX Gene—MYC-associated factor X
MKI67	Marker of proliferation Ki-67
MF	Molecular function
NCAPG	Non-SMC condensin I complex subunit G
NFY	Nuclear transcription factor Y
PCa	Prostate cancer
POPs	Persistent organic pollutants
PPI	Protein–protein interaction
PSA	Prostate-specific antigen
RNS	Reactive nitrogen species
ROS	Reactive oxygen species
RRM2	Ribonucleotide reductase regulatory subunit M2
STRING	Search tool for the retrieval of interacting genes/proteins
TCGA-PRAD	The Cancer Genome Atlas Prostate Adenocarcinoma
TF	Transcription factor
TPM	Transcript per million
TPX2	Targeting protein for Xenopus kinesin-like protein 2
FGFR2	Type 2 fibroblast growth factor receptor
UBE2C	Ubiquitin-conjugating enzyme E2 C
UCSC-TFBS	University of California, Santa Cruz–transcription factor binding sites
