# Supplementary material for: Endocrine Disrupting Chemicals Influence Hub Genes Associated with Aggressive Prostate Cancer
Source: Int J Mol Sci. 2023 Feb 6;24(4):3191. doi: 10.3390/ijms24043191 (PMC9959535; doi:10.3390/ijms24043191)
Supplement: Supplementary file 1 [file ijms-24-03191-s001.zip › ijms-2140666-supplementary.pdf]

Supplementary Materials

Table S1: Gene ontology and pathway enrichment analysis of **260 downregulated** overlapping genes' function in PCa. Top five in biological process (BP), cell component (CC), molecular function (MF), and KEGG.

	BP	Term	Count	P-Value	Genes
1	GO:0051301	Cell division	18	1.65×10^{-11}	CDCA2, CDCA3, UBE2C, CDCA5, CDCA8, NCAPG, BUB1B, ZWINT, AURKA, CCNA2, CCNB2, TPX2, CENPF, CCNB1, NUF2, CKS2, CDK1, BIRC5
2	GO:0032355	Response to estradiol	6	8.26×10^{-6}	CCNB1, KIF4A, STMN1, NUF2, WDR62, AURKA
3	GO:0000281	Mitotic cytokinesis	6	2.85×10^{-5}	ANLN, RACGAP1, KIF4A, STMN1, NUSAP1, BIRC5
4	GO:0044772	Mitotic cell cycle phase transition	4	2.4×10^{-4}	CCNA2, CCNB2, CCNB1, CKS2
5	GO:0030855	Epithelial cell	6	4.45×10^{-4}	CASP8, MYC, CASP3, DNMT3B, CYP1B1, EZH2
	**CC**	**Term**	**Count**	**P-Value**	
1	GO:0005829	Cytosol	56	4.7×10^{-8}	RPL30, MPI, BUB1B, IKZF1, LMNB1, CASP8, RPS18, CASP3, STMN1, NUF2, RAC3, TK1, MEN1, SREBF1, CKAP2L, RBM18, CCNA2, SAPCD2, BIRC5, BIRC2, CDCA2, AHCY, CDCA3, CDCA5, NCAPG, CDCA8, HMMR, WDR62, AURKA, HSP90B1, NT5C, CCNB2, CCNB1, RACGAP1, HAO1, RPS2, POLR2H, SPTBN2, NPM1, RRM2, UBE2C, ODF2, PTK6, ZWINT, GNMT, ANLN, TPX2, CENPF, KIF4A, ASB9, CDK1, GNAS, BAX, GRB2, FGF13, RPS21
2	GO:0005654	Nucleoplasm	43	9.98×10^{-7}	TOP2A, CDCA2, CDCA5, CDCA8, TWIST1, IKZF1, MKI67, AURKA, LMNB1, REPIN1, CCNB1, CASP8, RACGAP1, ZIC2, RPS18, MYC, CASP3, NUF2, SNAPC4, DNMT3B, RPS2, POLR2H, MEN1, SREBF1, NPM1, UBE2C, RBM18, CBX2, PTK6, IMP4, ZWINT, CCNA2, ANLN, TPX2, CENPF, SAPCD2, KIF4A, CDK1, BIRC5, GRB2, RPS21, ABCG2, EZH2
3	GO:0030496	Midbody	9	5.52×10^{-6}	ANLN, CENPF, RACGAP1, KIF4A, CDK1, CDCA8, BIRC5, HSP90B1, AURKA
4	GO:0000775	Chromosome, centromeric region	6	2.11×10^{-5}	TOP2A, CENPF, CDCA5, NUF2, CDCA8, BIRC5
5	GO:0000307	Cyclin-dependent protein kinase holoenzyme complex	74	5.34×10^{-5}	CCNA2, CCNB2, CCNB1, CDK1, CKS2
	**MF**	**Term**	**Count**	**P-Value**	
1	GO:0003682	Chromatin binding	11	2.8×10^{-4}	TOP2A, SREBF1, NPM1, CENPF, CDCA5, CBX2, CDK1, CKS2, DNMT3B, EZH2, MEN1
2	GO:0005515	Protein binding	85	1.86×10^{-3}	TOP2A, RPL30, BUB1B, IKZF1, HTR4, MKI67, LMNB1, SEC61A1, CASP8, RPS18, CDH1, MYC, CASP3, STMN1, NUF2, NUSAP1, TNFSF10, PBK, DNMT3B, CYP1B1, RAC3, TK1, MEN1, ABCC4, SREBF1, PAQR6, ARRDC1, IMP4, CPT1B, F5, CCNA2, SFRP4, SAPCD2, CKS2, AGTR1, BIRC5, BIRC2, ABCG2, AHCY, CDCA3, CDCA5, LHB, NCAPG, CDCA8, TWIST1, KLK3, HMMR, WDR62, STYXL1, AURKA, HSP90B1, CCNB2, CCNB1, ERBB3, RACGAP1, RAB26, RPS2, MPZL1, NPM1, RRM2, EGF, UBE2C, GOLM1, ODF2, CBX2, RASSF7, RAB39B, PTK6, ZWINT, GNMT, TPX2, CENPF, MLPH, KIF4A, ASB9, CDK1, GNAS, BAX, GRB2, RPL22L1, SHBG, FGF13, RPS21, CRB3, EZH2
3	GO:0019901	Protein kinase binding	10	6.4×10^{-6}	CCNA2, SREBF1, TPX2, NPM1, CCNB1, RACGAP1, CKS2, RAC3, GRB2, AURKA
4	GO:0008017	Microtubule binding	7	6.9×10^{-6}	TPX2, CENPF, RACGAP1, KIF4A, NUSAP1, BIRC5, FGF13
5	GO:0047485	Protein N-terminus binding	5	4.1×10^{-3}	NPM1, RPS21, BIRC2, ZWINT, MEN1
	**KEGG**	**Term**	**Count**	**P-Value**	
1	hsa05200	Pathways in cancer	16	1.3×10^{-5}	EGF, KLK3, HSP90B1, CCNA2, CASP8, CDH1, MYC, CASP3, CKS2, AGTR1, GNAS, BAX, BIRC5, RAC3, GRB2, BIRC2
2	hsa04115	p53 signaling pathway	7	2.3×10^{-5}	CCNB2, CCNB1, RRM2, CASP8, CASP3, CDK1, BAX
3	hsa05215	Prostate cancer	4	4.2×10^{-5}	EGF, KLK3, GRB2, HSP90B1
4	hsa04215	Apoptosis - multiple species	5	1.2×10^{-4}	CASP8, CASP3, BAX, BIRC5, BIRC2

5	hsa05206	MicroRNAs in cancer	6	3.8×10^{-3}	ERBB3, MYC, CASP3, CDCA5, STMN1, DNMT3B, CYP1B1, GRB2, EZH2

Table S2: Gene ontology and pathway enrichment analysis of **109 up-regulated** overlapping genes' functions in PCa. Top five in biological process (BP), cell component (CC), molecular function (MF), and KEGG.

	**BP**	**Term**	**Count**	**P-Value**	**Genes**
1	GO:0016486	Peptide hormone processing	4	1.7×10^{-4}	PCSK1N, ECE2, PCSK6, PLA2G7
2	GO:0015909	Long-chain fatty acid transport	3	1.21×10^{-2}	FABP5, APOE, SLC27A4
3	GO:0006111	Regulation of gluconeogenesis	3	1.27×10^{-2}	ZNF692, OGT, FBP1
4	GO:0001666	Low-density lipoprotein particle remodeling	3	1.27×10^{-2}	PLA2G2A, APOE, PLA2G7
5	GO:0019722	Calcium-mediated signaling	8	1.44×10^{-2}	BHLHA15, TRPM8, NCALD, CAMKK2, TRPM4
	**CC**	**Term**	**Count**	**P-Value**	
1	GO:0070062	Extracellular exosome	48	2.57×10^{-6}	RAB3B, SPON2, COPA, STEAP4, HPGD, IDUA, ACSM1, CPNE7, COL12A1, HPN, NEDD4L, TXN, EVPL, SLC4A4, THBS4, COMP, C4A, GMDS, TUBB3, EPCAM, MYO6, PABPC1L2B, APOE, TSPAN1, GALNT7, CYP2J2, LRRC26, SLC13A3, MARCKSL1, GDF15, PLA2G2A, TMEM132A, BGN, TMC5, NME1, GGCT, ALDH1A3, BCAM, FOLH1, TUBB2A, FABP5, FASN, SMS, RAB17, SMPDL3B, FBP1, SLC27A2, CFB
2	GO:0043025	Neuronal cell body	11	1.03×10^{-2}	C4A, TUBB3, HPN, EEF1A2, RGS10, KCNN2, RAB17, APOE, MAPK8IP2, RAP1GAP, TRPM4
3	GO:0016323	Basolateral plasma membrane	8	1.25×10^{-3}	SLC13A3, HPGD, EPCAM, CLDN8, RAB17, SLC4A4, SLC26A6, SLC19A1
4	GO:0005886	Plasma membrane	68	1.64×10^{-2}	EPHB6, RAB3B, KCNG3, STEAP4, CPNE7, CBLC, ECE2, PCSK6, SLC4A4, FGFRL1, C4A, SMPD2, SLC13A3, DAPK1, RALGAPA2, VSTM2L, ATAD3B, PTP4A3, BCAM, FOLH1, CLDN3, TRAF4, CLDN8, GPR160, CHMP4C, TRIB3, TLCD1, SMPDL3B, ZP3, SLC27A2, CFB, SLC27A4, COLEC12, SHC2, STX19, HPN, SLC43A1, IFI6, NEDD4L, UAP1, ADRB1, EPCAM, MYO6, LRIG1, REPS2, KCNN2, TRPM8, APOE, TSPAN1, SLC19A1, TRPM4, OR51E1, TMEM184A, MARCKSL1, OR51E2, PLA2G2A, PILRB, BAIAP3, SDK1, AMACR, FABP5, FASN, RGS10, PLXNB3, RAB17, OGT, RAMP1, SLC26A6
5	GO:0031012	Extracellular matrix	8	1.95×10^{-2}	COLEC12, COMP, SPON2, LRIG1, BGN, COL9A2, APOE, ZP3
	**MF**	**Term**	**Count**	**P-Value**	
1	GO:0016491	Oxidoreductase activity	10	1.18×10^{-3}	ALDH1A3, ALDH3B2, HPGD, BDH1, FASN, CHDH, MICAL2, DECR2, PAOX, CBR3
2	GO:0004467	Long-chain fatty acid-CoA ligase activity	3	1.21×10^{-2}	ACSM1, SLC27A2, SLC27A4
3	GO:0005324	Long-chain fatty acid transporter activity	3	1.73×10^{-2}	FABP5, SLC27A2, SLC27A4
4	GO:0005198	Structural molecule activity	9	1.92×10^{-2}	COPA, CLDN3, KRT17, CLDN8, APOE, MAPK8IP2, EVPL
5	GO:0005310	Dicarboxylic acid transmembrane transporter activity	5	2.25×10^{-2}	SLC13A3, SLC25A10
	**KEGG**	**Term**	**Count**	**P-Value**	
1	hsa05200	Pathways in cancer	31	1.53×10^{-2}	IDUA, ACSM1, GCNT1, UAP1, PLA2G7, ACACA, NME1-NME2, SMPD2, ALDH3B2, GMDS, CBS, CBR3, MCCC2, GALNT7, CYP2J2, ENTPD5, PLA2G2A, B3GAT1, PYCR1, MBOAT2, B3GALT6, NME1, GGCT, ALDH1A3, FOLH1, AMACR, BDH1, FASN, CHDH, SMS, FBP1
2	hsa04020	Calcium signaling pathway	7	1.54×10^{-2}	COLEC12, COMP, TUBB2A, HLA-DMB, SFTPA2, TUBB3, THBS4
3	hsa04270	Metabolic pathways	4	1.90×10^{-2}	MARCKSL1, HLA-DMB, EEF1A2, MAPK12
4	hsa04015	Rap1 signaling pathway	7	2.33×10^{-2}	COLEC12, COMP, TUBB2A, HLA-DMB, SFTPA2, TUBB3, THBS4
5	hsa04014	Ras signaling pathway	5	2.84×10^{-2}	AMACR, PEX10, DECR2, SLC27A2, PAOX

Table S3: Functional roles of 12 hub genes in biological processes are closely connected to PCa.

Regulated	No.	Symbol	Name	Function
Up-Regulated	1	NCAPG	Non-SMC Condensin I Complex Subunit G	Connected with NVAPG and functions as a target of miR-99a-3p in PCa cells, overexpression is connected to castrate-resistant prostate cancers (CRPC), in which a sustained AR signal is yet considered the primary cause of CRPC [61].
	2	MKI67	Marker of proliferation Ki-67	It is connected with two markers for PCa: PSA for tumor cell differentiation and KI-67 for tumor cell proliferation and the epithelial-mesenchymal transition [62].
	3	TPX2	Targeting protein for Xenopus kinesin-like protein 2	TPX2 expression in PCa tissues was increased compared with normal tissues. TPX2 is a microtubule-connected protein linked to mitosis and spindle assembly and targeting TPX2 is a strategy of PCa [63,64].
	4	CCNA2	Cyclin A2	CCNA2 contributes to PCa invasion by modulating the expression of metalloproteinase 2 (MMPs), MMP9, and vascular endothelial growth factor (VEGF) and by interacting with AR [65].
	5	CCNB1	Cyclin B1	Higher levels of CCNB1 in PCa cells may be a beneficial effect of polyploidy and a prognostic biomarker for chemotherapy [66].
Down-Regulated	6	CDK1	Cyclin Dependent Kinase 1	CDK1 controlled mitochondrial metabolism for bioenergetics needed for tumor cell survival and overexpression of CDK1 associated with poor prognosis and metastasis in PCa [67].
	7	CCNB2	Cyclin B2	CCNB2 particularly binds CDC2 to improve cell migration which is connected to the development of CRPC and also plays a critical part in transforming growth factor beta-mediated cell cycle control [68].
	8	UBE2C	Ubiquitin-conjugating enzyme E2C	UBE2C played an essential function in the pathway of PCa by the WNT-β-catenin signaling pathway and NOTCH signaling pathway [69].
	9	AURKA	Aurora kinase A	AURKA considered to be a possible prognostic biomarker for the progression of high-risk small-cell PCa [70].
	10	BUB1B	BUB1 mitotic checkpoint serine/threo	BUB1B is a critical mitotic checkpoint kinase and identified as the top-scoring kinase by RNA interaction [71].

			nine kinase B	
	11	CENPF	Centromere protein F	CENPF encodes a protein that associates with the G2 phase, cell growth, protein synthesis, and in the centromere-kinetochore complex and chromosomal segregation, it is related to aggressive prostate cancer [72].
	12	RRM2	Ribonucleotide reductase regulatory subunit M2	RRM2 restricts the rate of DNA synthesis and repair. It was believed to be a biomarker in PCa low-risk patients [73,74].

Table S4: Hub Genes for highly DEGs classified in Cytohubba plugin of Cytoscape in the PPI network according to four topological analysis techniques, including MCC, DNMC, Degree, and EPC (Up-regulated five genes: NCAPG, MKI67, TPX2, CCNA2, CCNB1, Down-regulated seven genes: CDK1, CCNB2, AURKA, UBE2C, BUB1B, CENPF, RRM2).

**Rank methods by Topological methods**	**MCC***	**DNMC***	**Degree**	**EPC***
**Top 20 genes**	**NCAPG**	**NCAPG**	**NCAPG**	**NCAPG**
	HMMR	KIF4A	RACGAP1	KIF4A
	RACGAP1	RACGAP1	BIRC5	MYC
	NUSAP1	BIRC5	MYC	CKS2
	KIF4A	MYC	**MKI67**	**MKI67**
	**MKI67**	**MKI67**	**TPX2**	**TPX2**
	**TPX2**	**TPX2**	TP53	TP53
	CDCA8	TP53	PBK	ANLN
	PBK	**CDK1**	**CDK1**	PBK
	**CDK1**	CENPN	CENPN	**CDK1**
	**CCNB2**	**CCNB2**	**CCNB2**	ECT2
	CDCA5	EZH2	EZH2	**CCNB2**
	**AURKA**	**AURKA**	**AURKA**	EZH2
	**UBE2C**	**UBE2C**	**UBE2C**	**AURKA**
	**CCNA2**	**CCNA2**	**CCNA2**	**UBE2C**
	**CCNB1**	**CCNB1**	**CCNB1**	**CCNA2**
	**BUB1B**	**BUB1B**	**BUB1B**	**CCNB1**
	**CENPF**	**RRM2**	**RRM2**	**BUB1B**
	**RRM2**	**CENPF**	**CENPF**	**RRM2**
	TOP2A	TOP2A	TOP2A	**CENPF**

*MCC: Maximal Clique Centrality, DNMC: Degree, Density of Maximum Neighborhood Component, and EPC: Degree and Edge percolated component.

Table S5: The Gleason scores risk-based categorization (Low, Medium, and High risk).

Grade group	Gleason score	Characteristic	The research categorization
1	6	Less aggressive<br>low risk	Low Risk [32, 33]
2	(3+4) = 7	Slightly aggressive<br>Low to intermediate risk	Medium Risk [32, 33]
3	(4+3) = 7	Moderate aggressive<br>intermediate risk	
4	8	Aggressive<br>High risk	High risk [32, 33]
5	9-10	Highly aggressive<br>High risk	

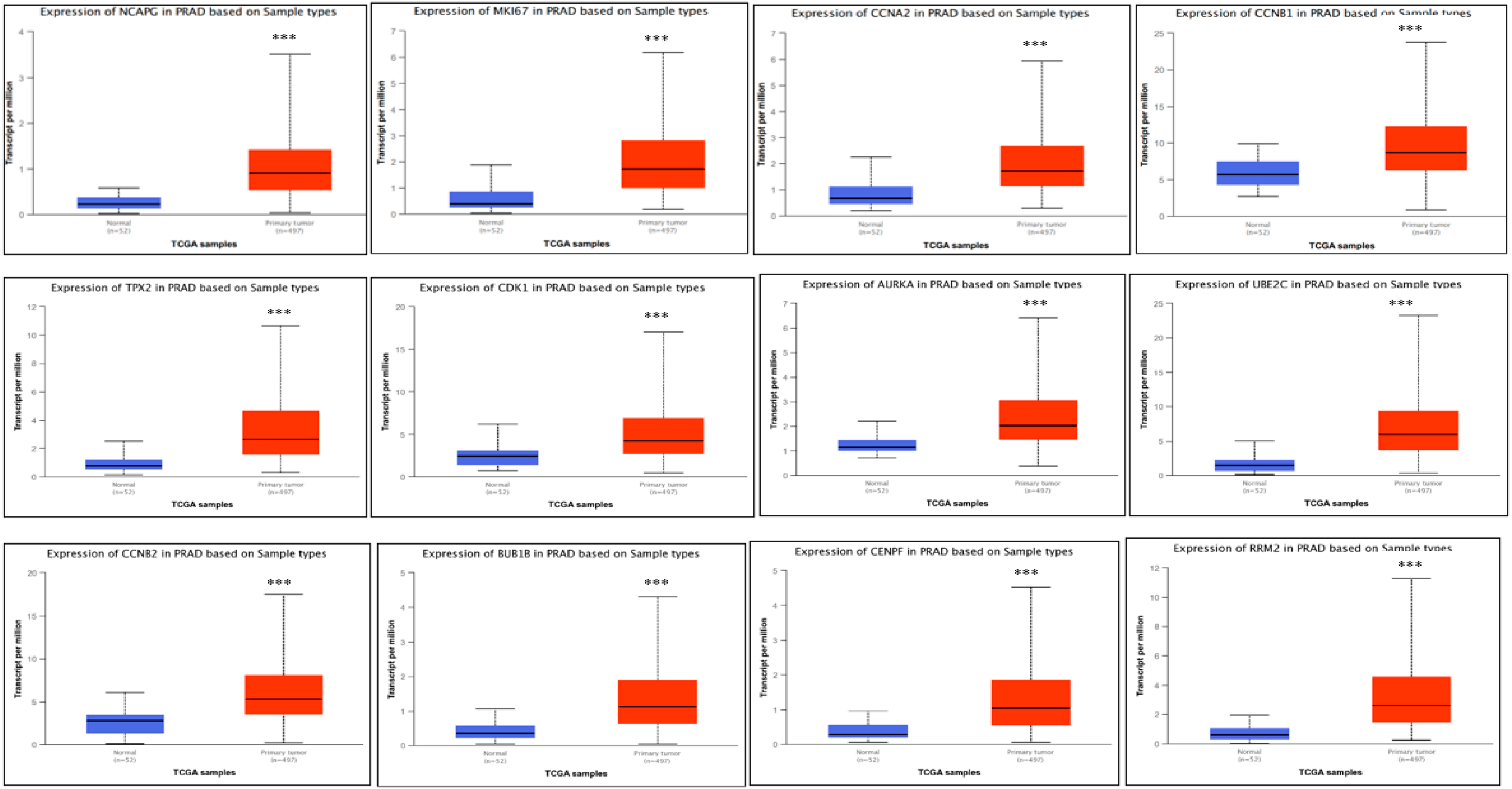

Figure S1: Box whisker plots indicate the twelve hub gene expressions in PCa samples. Five Up-Regulated Hub Genes (NCAPG, MKI67, CCNA2, CCNB1, TPX2) and seven Down-Regulated Hub Genes (CDK1, CCNB2, AURKA, UBE2C, BUB1B, CENPF, RRM2) were identified by MCODE and verified at the protein level by UALCAN, which came from the TCGA project. Data are mean ± SE. ***: p-value <0.001.

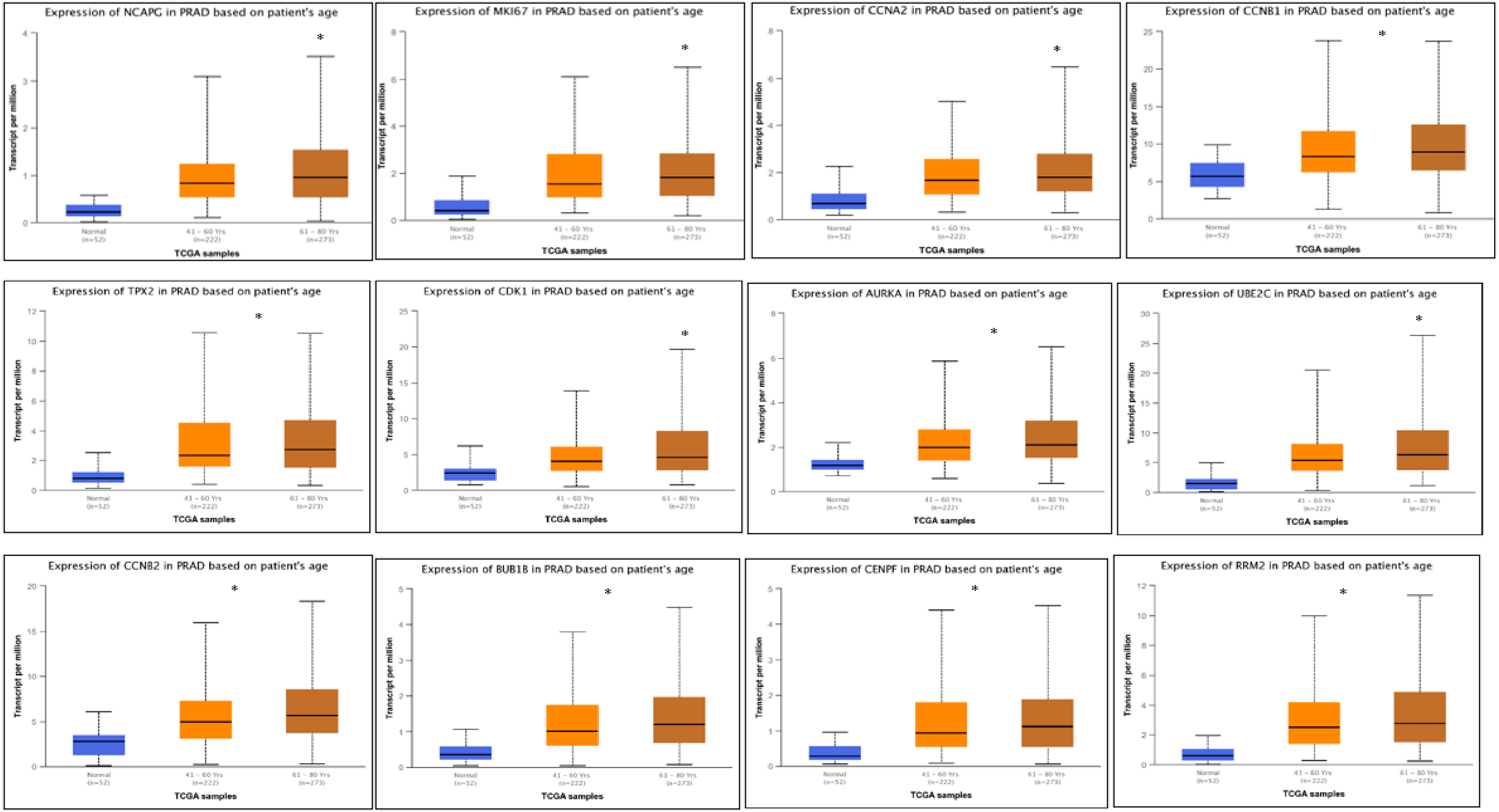

Figure S2: Box whisker plots indicate the expressions of twelve hub genes in PCa samples based on the age of patients. Five Up-Regulated Genes (NCAPG, MKI67, CCNA2, CCNB1, TPX2) and seven Down-Regulated Genes (CDK1, CCNB2, AURKA, UBE2C, BUB1B, CENPF, RRM2) were identified by MCODE and verified at the protein level by UALCAN, which came from the TCGA project. Data are mean ± SE. *: p-value < 0.05.

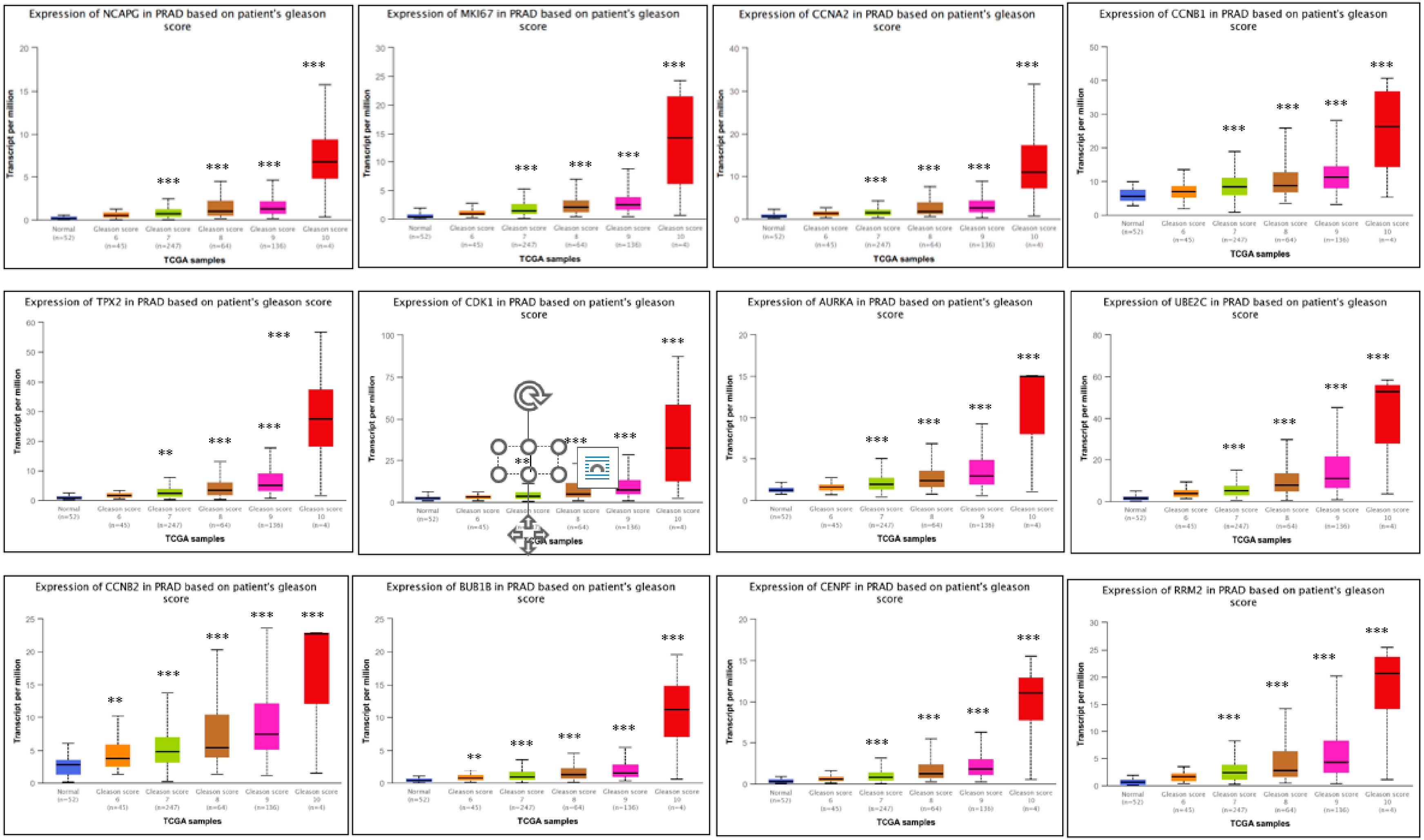

Figure S3: Box whisker plots indicate expressions of twelve hub genes' in PCa patients' samples with different Gleason scores. Five Up-Regulated Hub Genes (NCAPG, MKI67, CCNA2, CCNB1, TPX2) and seven Down-Regulated Hub Genes (CDK1, CCNB2, AURKA, UBE2C, BUB1B, CENPF, RRM2) were identified by MCODE and verified at the protein level by UALCAN, which came from the TCGA project. Data are mean ± SE. **: p-value <0.01; ***: p-value <0.001.

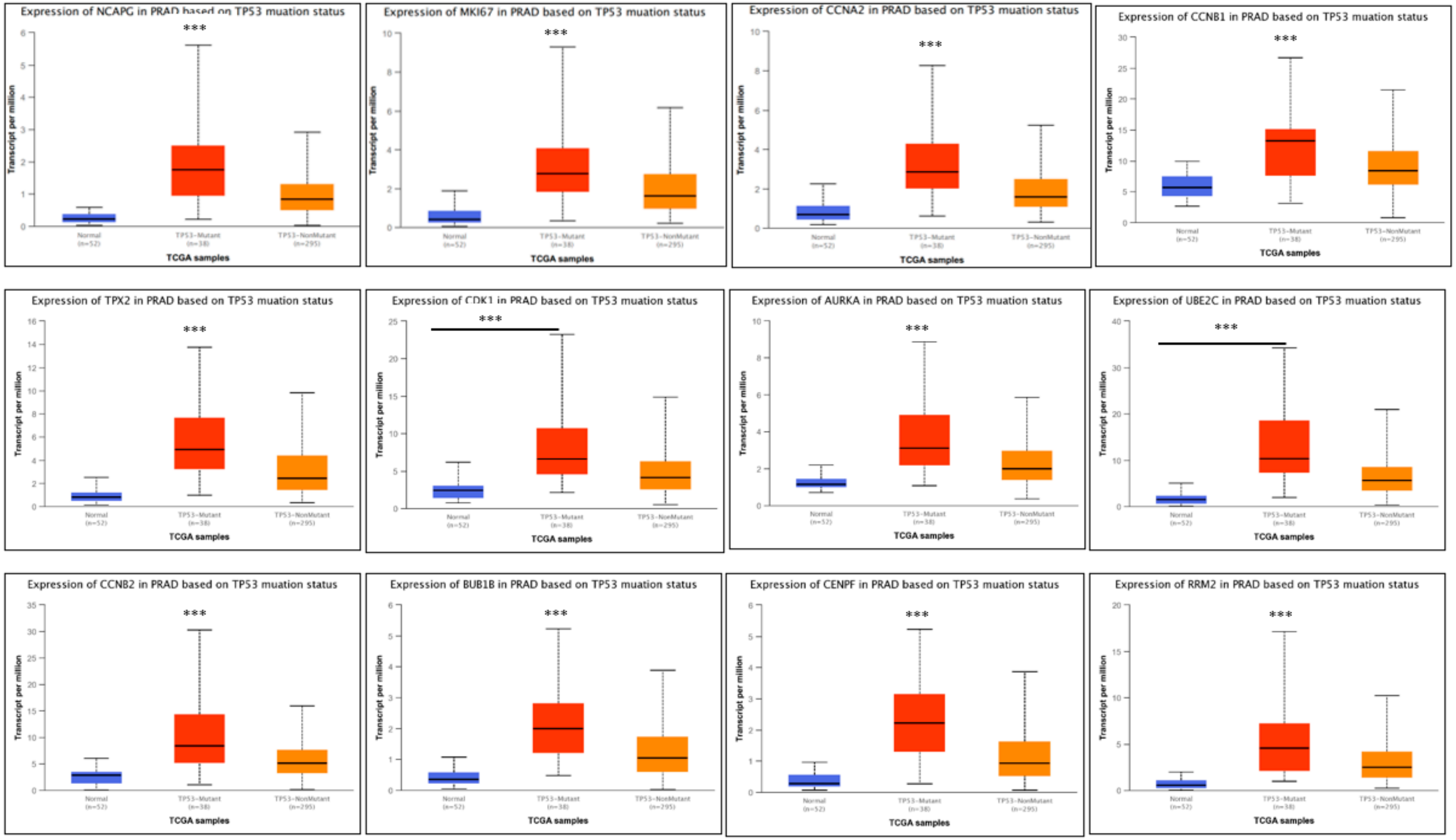

Figure S4: Box whisker plots indicate the expression of twelve hub genes in PCa samples with TP53 mutations.. Five Up-Regulated Hub Genes (NCAPG, MKI67, CCNA2, CCNB1, TPX2) and seven Down-Regulated Hub Genes (CDK1, CCNB2, AURKA, UBE2C, BUB1B, CENPF, RRM2) were identified by MCODE and verified at the protein level by UALCAN, which came from the TCGA project. Data are mean ± SE. Data are mean ± SE. ***: p-value <0.001.
